# Cellular APOBEC3A deaminase drives mutations in the SARS-CoV-2 genome

**DOI:** 10.1093/nar/gkac1238

**Published:** 2023-01-05

**Authors:** Yoshihiro Nakata, Hirotaka Ode, Mai Kubota, Takaaki Kasahara, Kazuhiro Matsuoka, Atsuko Sugimoto, Mayumi Imahashi, Yoshiyuki Yokomaku, Yasumasa Iwatani

**Affiliations:** Clinical Research Center, National Hospital Organization Nagoya Medical Center, Nagoya, Aichi 460-0001, Japan; Department of AIDS Research, Division of Basic Medicine, Nagoya University Graduate School of Medicine, Nagoya, Aichi 466-8550, Japan; Clinical Research Center, National Hospital Organization Nagoya Medical Center, Nagoya, Aichi 460-0001, Japan; Clinical Research Center, National Hospital Organization Nagoya Medical Center, Nagoya, Aichi 460-0001, Japan; Clinical Research Center, National Hospital Organization Nagoya Medical Center, Nagoya, Aichi 460-0001, Japan; Department of Respiratory Medicine, Division of Internal Medicine, Nagoya University Graduate School of Medicine, Nagoya, Aichi 466-8550, Japan; Clinical Research Center, National Hospital Organization Nagoya Medical Center, Nagoya, Aichi 460-0001, Japan; Clinical Research Center, National Hospital Organization Nagoya Medical Center, Nagoya, Aichi 460-0001, Japan; Clinical Research Center, National Hospital Organization Nagoya Medical Center, Nagoya, Aichi 460-0001, Japan; Clinical Research Center, National Hospital Organization Nagoya Medical Center, Nagoya, Aichi 460-0001, Japan; Clinical Research Center, National Hospital Organization Nagoya Medical Center, Nagoya, Aichi 460-0001, Japan; Department of AIDS Research, Division of Basic Medicine, Nagoya University Graduate School of Medicine, Nagoya, Aichi 466-8550, Japan

## Abstract

The number of genetic variations in the SARS-CoV-2 genome has been increasing primarily due to continuous viral mutations. Here, we report that the human APOBEC3A (A3A) cytidine deaminase plays a critical role in the induction of C-to-U substitutions in the SARS-CoV-2 genome. Bioinformatic analysis of the chronological genetic changes in a sequence database indicated that the largest UC-to-UU mutation signature, consistent with APOBEC-recognized nucleotide motifs, was predominant in single-stranded RNA regions of the viral genome. In SARS-CoV-2-infected cells, exogenous expression of A3A but not expression of other APOBEC proteins induced UC-to-UU mutations in viral RNA (vRNA). Additionally, the mutated C bases were often located at the tips in bulge or loop regions in the vRNA secondary structure. Interestingly, A3A mRNA expression was drastically increased by interferons (IFNs) and tumour necrosis factor-α (TNF-α) in epithelial cells derived from the respiratory system, a site of efficient SARS-CoV-2 replication. Moreover, the UC-to-UU mutation rate was increased in SARS-CoV-2 produced from lung epithelial cells treated with IFN-ß and TNF-α, but not from CRISPR/Cas9-based A3A knockout cells. Collectively, these findings demonstrate that A3A is a primary host factor that drives mutations in the SARS-CoV-2 RNA genome via RNA editing.

## Graphical Abstract

**Graphical Abstract:**
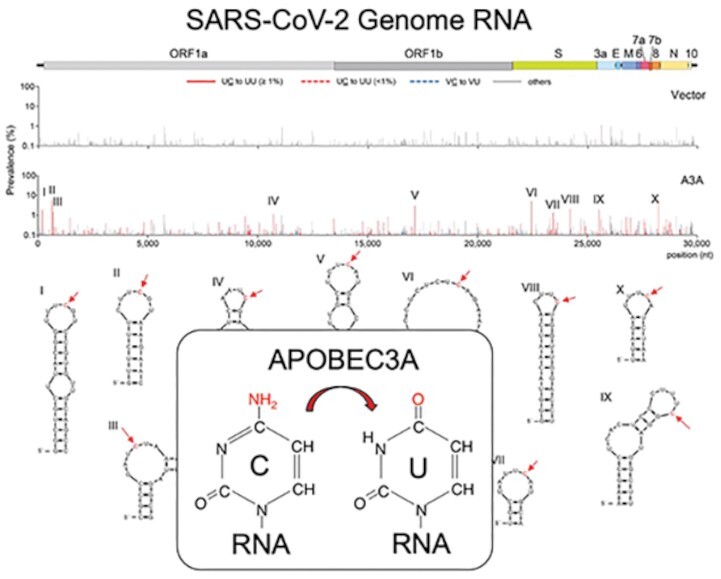
Endogenous and exogenous expression of APOBEC3A cytidine deaminase induced UC-to-UU mutations in the SARS-CoV-2 genome, which were often located at the tips in loop regions in the viral genomic RNA.

## INTRODUCTION

Coronavirus disease 2019 (COVID-19), which is caused by severe acute respiratory syndrome coronavirus 2 (SARS-CoV-2), has spread rapidly worldwide since December 2019 (1,2). Concomitantly, genomic variation in SARS-CoV-2 has increased, with different variants of SARS-CoV-2 lineages emerging in multiple geographical regions of the world. Certain SARS-CoV-2 lineages have been designated variants of concern (VOCs) because of their increased transmissibility, related disease severity and immune escape properties. These genetic changes, characteristically comprising insertions, deletions and substitutions (3), have been reported in various genomic surveillance databases, such as the Global Initiative on Sharing All Influenza Data (GISAID) EpiCoV (referred to as the ‘GISAID’ in this report), and are regarded as outcomes of viral selection for propagation in hosts. Most genetic changes in SARS-CoV-2 that negatively affect viral replication are likely unsustained, whereas a small proportion that do not affect or benefit viral infectivity, immune escape, or tolerance to antiviral treatments have been observed in the database sequences (4).

The primary sources of substitutions in the SARS-CoV-2 genome arise from viral replication errors during error-prone RNA-dependent polymerization, or ribonucleotide misincorporation. These events occur despite the reduction in the error magnitude by the viral proofreading exoribonuclease (ExoN) in the N-terminal domain of nonstructural protein 14 (nsp14), which is commonly encoded in the genomes of *Coronaviridae* but not in those of other RNA viruses (reviewed in ref. (5)). Additionally, an early study based on public RNA transcriptome datasets containing data on bronchoalveolar lavage fluid from COVID-19 patients suggested that host deaminases may play roles in the increased genetic variation in the SARS-CoV-2 RNA genome (6). In this study, two potential host deaminases, APOBEC polynucleotide cytidine (C) deaminases and adenosine (A) deaminase RNA specific (ADAR), were assessed for their potential effects on two types of dinucleotide mutation signatures, UC-to-UU (U, uridine) and AA-to-AG (G, guanosine) substitutions, respectively. Furthermore, sequence analysis of datasets available from GISAID suggested that the nucleotide changes in the SARS-CoV-2 genome during the initial months of the 2020 pandemic primarily involved C-to-U mutations, likely driven by a host APOBEC-like editing process (7,8). However, it remains unclear whether cellular APOBEC cytidine deaminases directly play a critical role in driving SARS-CoV-2 genome mutations.

The APOBEC3 (A3) family comprises seven members (A, B, C, D, F, G and H) in primates, and these members exhibit potent antiviral activity against retroviruses and retroelements (9,10). Before the discovery of the A3 family, the A3 family member APOBEC3A (A3A) was initially identified as an APOBEC1 (A1) analogue called phorbolin-1, which was expressed in primary keratinocytes treated with phorbol 12-myristate-1-acetate (PMA) (11). In addition, the A3 family members, especially A3A and APOBEC3B (A3B), are intrinsic mutators of chromosomal DNA, although how these enzymes contribute to the accumulation of mutations driving cancer formation is still under debate (12–14). Generally, A3A is detected in myeloid-lineage cells in the blood, lymph nodes and lung tissues (15,16). In macrophages, A3A expression is greatly increased by type I interferons (IFNs), tumour necrosis factor-α (TNF-α) or hypoxia (17,18). A3G, a potent inhibitor of retrovirus replication, is expressed predominantly in lymphoid and myeloid cells (9,10,15). These APOBEC proteins, including A1 and activation-induced cytidine deaminase (AID), commonly exhibit polynucleotide cytidine deaminase activity preferentially toward single-stranded DNA (ssDNA) (10). In addition, previous studies have shown that A1 (19–21), A3A (14,18,22) and A3G (23) catalyse cytosine deamination in single-stranded RNA (ssRNA), i.e. RNA editing, although whether the APOBEC3G (A3G) deaminate RNA substrates remains controversial. Therefore, it has been assumed that the APOBECs with RNA editing activity might contribute to driving SARS-CoV-2 genome mutations. However, whether any APOBECs are directly involved in the induction of C-to-U mutations during SARS-CoV-2 replication has not been established.

In this study, we performed bioinformatic analysis of SARS-CoV-2 genome sequences in GISAID to re-evaluate whether the APOBEC-like signature of UC-to-UU mutations is still continuously generated in the genome. In addition, we investigated the effects of the cellular expression of APOBEC cytidine deaminases on the induction of UC-to-UU mutations in the viral RNA (vRNA) genome using live viruses. We found that endogenous and exogenous expression of A3A in cells induced UC-to-UU mutations in the SARS-CoV-2 genome. The mutation hotspots mediated by A3A were often located at the tips in the loop regions in the viral genome. Our findings in this study suggest that A3A is a major cellular factor that increases genetic variations in the SRAS-CoV-2 RNA genome.

## MATERIALS AND METHODS

### Cells and viruses

Human embryonic kidney 293T (293T) cells stably expressing human angiotensin converting enzyme 2 (ACE2) and transmembrane serine protease 2 (TMPRSS2) (293T/ACE2-TMPRSS2 cells, hereafter referred to as 293AT cells) were purchased from GeneCopoeia, Inc. Calu-3 cells (human lung-derived adenocarcinoma [epithelial-like] cell line) and Vero E6 cells were obtained from the American Type Culture Collection (ATCC), and Vero E6 cells stably expressing human TMPRSS2 (Vero E6/TMPRSS2 cells), A549 human adenocarcinoma (epithelial-like) cells, and MRC-5 foetal lung fibroblast cells were obtained from the Japanese Collection of Research Bioresources Cell Bank. 293T, 293AT, Vero E6, Vero E6/TMPRSS2, A549, and MRC-5 cells were maintained in Dulbecco's modified Eagle's medium (DMEM) (Sigma–Aldrich) supplemented with 10% foetal bovine serum (FBS), penicillin (100 U/ml) and streptomycin (100 μg/ml) (Thermo Fisher Scientific) (hereafter referred to as DMEM GM). Calu-3 cells were cultured in DMEM/nutrient mixture F-12 (Thermo Fisher Scientific) containing 10% FBS, penicillin (100 U/ml) and streptomycin (100 μg/ml) (hereafter referred to as DMEM/F-12 GM). Normal primary human small airway epithelial (SAE) cells and normal primary human lobar bronchial epithelial (LBE) cells were obtained from ATCC. Human nasal epithelial primary cells (HNEpCs) and human type II alveolar epithelial (AT2) cells were purchased from PromoCell and Accegen Biotechnology, respectively. Primary epithelial cells were maintained in airway cell basal medium (ATCC) supplemented with the components of a bronchial epithelial cell growth kit (ATCC), penicillin (10 U/ml) and streptomycin (10 μg/ml) according to the ATCC-recommended culture protocols.

Primary monocyte-derived macrophages (MDMs) were prepared from human peripheral blood mononuclear cells (PBMCs) of healthy donors as described previously (24). In brief, monocytes were first isolated from PBMCs using a Classical Monocyte Isolation Kit (Miltenyi Biotec). CD14^+^ cells were plated at 5 × 10^5^ cells/well in RPMI 1640 medium (Sigma–Aldrich) supplemented with penicillin (100 U/ml) and streptomycin (100 μg/ml) for 3 h prior to the addition of 10% FBS and 10 ng/ml macrophage colony stimulating factor (PeproTech). Adherent cells were cultured for 7 dys and used as MDMs.

The virus strain used in this study was SARS-CoV-2 B.1.1 (Pango Lineage B.1.1, GISAID EPI_ISL_568558), which was isolated from a nasal swab sample of a patient in the Nagoya Medical Center, Japan, and then propagated in Vero E6 cells (25). Virus propagated in Vero E6 cells was stored at –80°C for use in subsequent experiments. To determine the virus titre, Vero E6/TMPRSS2 cells cultured in 96-well plates were incubated with 100 μl of serially diluted (twofold) virus in DMEM GM for 1 h at 37°C. The infected cells were then washed once with 100 μl of prewarmed fresh DMEM GM and incubated in fresh DMEM GM for 2 days at 37°C. The cytopathic effect was evaluated by microscopy. The median tissue culture infectious dose (TCID_50_) was determined by the Reed and Muench method (26). For analysis of vRNA, supernatants were first clarified by centrifugation at 750 × g for 10 min and were then filtered through a 0.45-μm-pore membrane (Merck Millipore).

### Plasmids and transfection

The expression plasmids for human AID with a C-terminal Myc-HIS tag were constructed in the pcDNA 3.1 (–) vector (Thermo Fisher Scientific). The expression plasmids for human A3A with a C-terminal Myc-HIS tag in the pcDNA 3.1 (–) vector and of A1 containing a C-terminal HA tag in the pCASSG vector, were obtained from Dr Klaus Strebel and Dr Terumasa Ikeda, respectively (27,28). The plasmids carrying A3B, APOBEC3C (A3C), APOBEC3D (A3D), APOBEC3F (A3F), and A3G in the pcDNA 3.1 (–) vector and the plasmid carrying APOBEC3H (A3H) (haplotype II) in the pTR600 vector were prepared as previously described (29–31). Notably, there was a technical difficulty in the preparation of the A3A and A3B expression plasmids that was caused partially by their high toxicity. In addition, the A3A and A3B fragments were prone to acquiring detrimental mutations during plasmid propagation in *Escherichia coli*. Therefore, we screened several *E. coli* strains that ultimately enabled us to prepare the A3A and A3B expression plasmids. Only the NEB Turbo strain (New England Biolabs) was used, and no obvious toxicity or detrimental mutations were observed during propagation.

For expression of the A1, A3 and AID proteins, each plasmid (0.5 μg) was transfected into 293AT cells in 12-well plates using FuGENE HD (Promega) according to the manufacturer's instructions. The cells were used for viral infection experiments thirty-six hours after transfection, and for immunoblot analysis of protein expression 48 h after transfection.

### Immunoblotting

Transfected cells were prepared in Laemmli buffer (Bio-Rad Laboratories) containing 2.5% 2-mercaptoethanol (2-ME). Proteins were separated on either 10% or 12% SDS–PAGE gels and transferred onto Immobilon-P membranes (Merck Millipore). The membranes were first probed with the appropriate primary antibodies. An anti-HIS tag monoclonal antibody (mAb), anti-HA tag mAb (both at a 1:2.5 × 10^3^ dilution) (Medical & Biological Laboratories Co.), anti-DYKDDDDK tag mAb (1:2 × 10^3^ dilution; for detecting the FLAG tag) (Fujifilm Wako Pure Chemical Co.), and anti-ß-tubulin antibody (1:10^3^ dilution; as the loading control) (Abcam) were used. The membranes were subsequently incubated with goat horseradish peroxidase (HRP)-conjugated anti-mouse IgG or anti-rabbit IgG (both 1:2 × 10^4^ dilution; Thermo Fisher Scientific) as secondary antibodies. Proteins were visualized by enhanced chemiluminescence using SuperSignal West Dura substrate (Thermo Fisher Scientific) with an ImageQuant LAS 4000 system (GE Healthcare Life Sciences).

### Deep sequencing and analysis of the viral genome

vRNA was extracted from 140 μl of culture supernatant using the QIAamp Viral RNA Mini Kit (QIAGEN) according to the manufacturer's protocol. Each of the isolated vRNAs was dissolved in 60 μl of RNase-free distilled water and stored at -80°C until sequencing analysis was performed. The next-generation sequencing (NGS) libraries (nt 55–29835, relative to the Wuhan reference sequence, hCoV-19/Wuhan/WIV04/2019, EPI_ISL_402124) were prepared with a QIAseq SARS-CoV-2 Primer Panel (QIAGEN) and a QIAseq FX DNA Library Kit (QIAGEN), according to the manufacturer's instructions. In brief, RNA samples were converted by reverse transcription into cDNA and enriched through two multiplex targeted PCRs with two different primer pools (pool1 and pool2). The enriched products in the two pool reactions were mixed and purified with an equal volume of AMPureXP beads (Beckman Coulter). The product concentrations were then quantified using a Qubit dsDNA High Sensitivity Assay Kit (Thermo Fisher Scientific) with a Qubit 2.0 fluorometer (Thermo Fisher Scientific). The purified products (250 ng) were subjected to fragmentation, end repair, and adaptor ligation for library preparation. After the series of reactions were completed, the libraries (average of ∼500 bp) were subjected to two rounds of size selection and purification (at 0.8 × and 1 × volumes, respectively) with AMPureXP beads (Beckman Coulter). The equimolar libraries were pooled (12 samples/pool), diluted to a final concentration of 10–13 pM, and then sequenced on the Illumina MiSeq platform using a MiSeq Reagent Kit v3 (300 cycles) (Illumina).

To identify minority mutations, the read data obtained through MiSeq were processed using our previously reported method (32) with slight modifications. In brief, the primer sequences were trimmed from the raw read sequences with the cutPrimers program (ver. 2.0) (33). The trimmed sequences were mapped to the reference sequence or a corresponding consensus sequence in the viral stock using the BWA-MEM algorithm in the BWA program (ver. 0.7.3a-r367) (34). Next, sequences with a mapping quality ≥ 60 were selected with the SAMtools program (ver. 0.1.18-r580) (35). To distinguish minority mutations from sequencing errors, a threshold was set at 1% relative abundance as the minority population, and error correction was carried out by filtering based on the per-site quality scores for each base (32). For this study, we examined minority mutations present in ≥ 1% of the population based on at least two read sequences. For experiments with the same virus isolates, we omitted shared mutations commonly observed in the isolates from the analyses. For bar graph showing the prevalence with C-to-U mutations throughout the viral genome, we grouped the mutational patterns in the dinucleotide context into three: UC-to-UU, VC-to-VU (V = not U), and others.

### IFN treatment under normoxic and hypoxic conditions

To measure A3A mRNA levels, the indicated cells were seeded in 12-well plates. After incubation for 24 h at 37°C, the cells were treated with human IFN beta 1a (IFN-ß) (1,000 U/ml) (PBL Assay Science) and/or recombinant human TNF-α (50 ng/ml) (Fujifilm Wako Chemicals) in DMEM GM under 5% CO_2_ for 18 h at 37°C (under normoxic conditions). For hypoxia treatment, immediately after adding IFN-ß and/or TNF-α, the cells were placed in a 1% O_2_ environment using a BIONIX-1 Hypoxic Culture Kit (Sugiyamagen) as previously reported (36). After incubation under hypoxic conditions for 18 h at 37°C, total mRNA was extracted from the cells.

For SARS-CoV-2 infection, Calu-3 cells (2 × 10^5^ cells/well) in 12-well plates were treated with or without IFN-ß (1,000 U/ml) and TNF-α (50 ng/ml) in DMEM/F-12 GM under 5% CO_2_ for 18 h at 37°C. After the cells were washed twice with 800 μl of fresh prewarmed DMEM/F-12 GM, they were incubated in fresh DMEM/F-12 GM for another 8 h and were then infected with SARS-CoV-2 B.1.1 at a high multiplicity of infection (MOI) of 0.5 for 4 h. The cells were then washed twice with DMEM/F-12 GM. The cells pretreated with IFN-ß and TNF-α (pretreatment condition, referred to as Pretreated) were further incubated in DMEM/F-12 GM for 72 h, and the culture supernatants were harvested for virus passaging and deep sequencing. The cells not pretreated with IFN-ß and TNF-α treatment before infection were incubated in the presence (posttreatment condition, referred to as Posttreated) or absence of IFN-ß (1000 U/ml) and TNF-α (50 ng/ml) (control) for 48 h before the cell culture supernatants were harvested. Each volume of harvested virus was passaged twice in the same manner (to passage 3, P3). Notably, SARS-CoV-2 production was delayed under the pretreatment condition compared with the posttreatment and control conditions, partially because SARS-CoV-2 is highly sensitive to IFN pretreatment (37–39). Therefore, virus-infected cells cultured under the pretreatment condition were incubated 24 h longer than those cultured under the posttreatment and control conditions.

### Quantitative reverse transcription–droplet digital PCR (RT–ddPCR) analysis

Total cellular RNA was isolated from cells cultured in 12-well plates by using ISOGEN-LS (Nippon Gene) according to the manufacturer's protocol. For quantification of the A3A, CD11b, CD68 and housekeeping ribonuclease P protein subunit p40 (RPP40) mRNA levels, RT–ddPCR was performed using a One-Step RT-ddPCR Advanced Kit for Probes (Bio-Rad Laboratories). In brief, a total volume of 22 μl per reaction was first prepared by mixing Supermix (5.5 μl), nuclease-free distilled water (1.1 μl), reverse transcriptase (2.2 μl), 300 mM dithiothreitol (1.1 μl), 55 ng of total RNA (5.5 μl), and either the A3A or RPP40 TaqMan gene expression probe-primer mixture (1.1 μl of the 20 × mixture). FAM-labelled A3A mRNA-specific (Hs00377444_m1), FAM-labelled CD11b mRNA-specific (Hs00355885_m1), FAM-labelled CD68 mRNA-specific (Hs00154355_m1) and VIC-labelled housekeeping RPP40 mRNA-specific (Hs01017007_m1) probe/primer mixtures (Thermo Fisher Scientific) were used. For each reaction solution, droplets were generated by loading 20 μl of the reaction mixture into a QX200 Droplet Digital PCR System (Bio-Rad Laboratories), and PCR amplification was performed with a ProFlex PCR system (Thermo Fisher Scientific) under the following thermal cycling conditions: reverse transcription for 60 min at 42°C, enzyme activation for 10 min at 95°C, 40 cycles of denaturation for 0.5 min at 95°C and a subsequent extension for 1 min at 60°C, and enzyme deactivation for 10 min at 98°C. After the PCRs, the reaction droplets were analysed with a QX200 Droplet Digital PCR system and QuantaSoft software (ver. 1.7) (Bio-Rad Laboratories). The ratios of the A3A, CD11b and CD68 mRNA copy numbers relative to that of RPP40 in equal amounts of total RNA were calculated.

### Database analysis of viral genome sequences and RNA secondary structures

The full genome (>29 000 nt) sequences (approximately 12.1 million sequences) downloaded on 24 July 2022, from the GISAID EpiCoV database (https://www.gisaid.org/) were used in this study. First, genome sequences with ambiguous bases or without a sampling date were excluded from the downloaded sequence file. Next, to attain a level of confidence for sequences targeting transmittable viruses, we extracted all the sequences in which the viral protein-coding region (nt 266–29 674 relative to the Wuhan reference sequence) was matched with one or more sequences. That is, genome sequences that did not have identical genotyped sequences in the database were excluded. In total, 2 051 393 sequence datasets were used for subsequent analyses (provided upon request). The metadata corresponding to the individual sequences, such as viral lineage information, were also obtained from the GISAID EpiCoV.

Nucleotide substitutions within individual genome sequences were identified as follows: First, sequences were mapped to the Wuhan reference sequence using the minimap2 program (ver. 2.17-r974) with the following options: -a -A 2 -O 24, 24 -E 2,2. Alignment data in CIGAR strings in the output SAM file were converted into a simple sequence alignment format as a FASTA file with an *in-house* program (provided upon request). Substitutions were counted based on the alignment information obtained with *in-house* alignment programs (provided upon request). Mononucleotide substitutions were simply counted by comparison with the reference sequence. To analyse substitutions at di- and trinucleotide motifs, substitutions were scored by considering the mono- or dinucleotide sequences immediately upstream of the substituted positions. In addition, to determine whether each substitution was synonymous or nonsynonymous, we examined whether the individual nucleotide substitution resulted in an amino acid change, considering the in-frame trinucleotide sequences flanking the substituted position. We referred to the RNA secondary structure of the SARS-CoV-2 genome, which was previously determined based on in vivo SHAPE data (40), and visualized it with the online application mFold web server (41) for verification.

## RESULTS

### Mutational signatures in the SARS-CoV-2 sequence database

**Figure 1.**
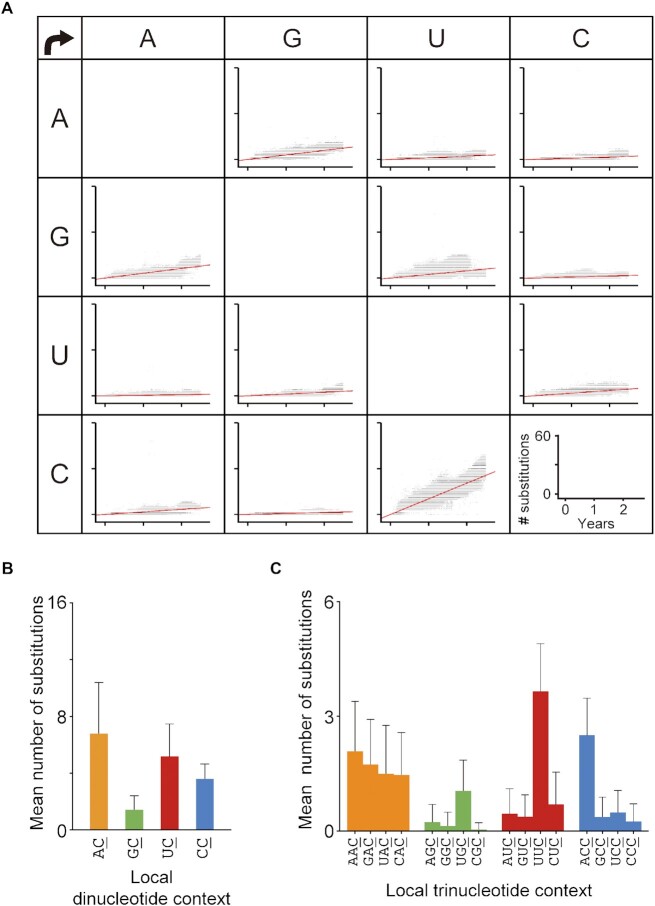
Chronological nucleotide changes observed in SARS-CoV-2 genome sequences in the GISAID database. (**A**) Chronological changes in the numbers of mononucleotide substitutions per genome were analysed for each SARS-CoV-2 sequence (*n* = 2 051 393) relative to the Wuhan reference strain. The number was plotted against the sample collection date corresponding to the sequence in the GISAID database. The red lines represent linear regression lines. The years ‘0’, ‘1’ and ‘2’ on the x-axis represent 2020, 2021 and 2022, respectively. (**B**, **C**) Di- and trinucleotide contexts where C-to-U substitutions were detected (underlined) were compared. The bar graphs show the means ± SDs (error bars) of the numbers of substitutions in all the sequences.

As an initial step towards identifying the mutator(s) involved in the vRNA mutations, we analysed chronological changes in the SARS-CoV-2 genome datasets (∼12.1 million sequences) reported in GISAID throughout the period of ∼2.5 years from December 2019 through June 2022. To increase the accuracy of the analysis, the genome sequences (2 051 393 sequences) were first extracted according to the following three criteria: (i) ambiguous bases were not included in the sequence; (ii) the sampling date was indicated and (iii) more than two matched sequences in the entire viral protein-coding region were found on the basis of a high probability of viral transmissibility. In the extracted sequence dataset, the rate of total mononucleotide substitutions was ∼26.9 nucleotides (nt) per year (nt/yr) in the 2.5-year period assessed, nearly identical to the real-time estimate reported by Nextstrain (30.6 nt/yr) (42). As observed in the early period, the C-to-U substitution rate remained high throughout the chronological period (10.2 nt/year), whereas its complementary G-to-A substitution rate was ∼3.1 nt/year, which was within the rate range of other mutations (Figure 1A). These results demonstrated that strand-biased C-to-U mutations in the SARS-CoV-2 genome continued to occur for 2.5 years. This chronological trend of C-to-U substitutions has also been observed in geographically distinct regions throughout the world (Supplementary Figure S1).

Next, focusing on the dinucleotide context, we found that the average numbers of genomic AC-to-AU and UC-to-UU substitutions were 6.8 and 5.2, respectively, higher than those of the other two dinucleotide substitutions (Figure 1B). Because the SARS-CoV-2 VOC Delta has a high AC-to-AU substitution rate, as detected in sequence datasets (34.3%), the overall AC-to-AU substitution rate was higher than that reported previously (7,8). This explanation is substantiated by evidence showing that only the UC-to-UU substitution increased over time, independent of the SARS-CoV-2 lineage (Supplementary Figure S2). In addition, mapping analysis of the mutation signatures in the vRNA secondary structure, which was previously determined by Manfredonia *et al.* (40) using SHAPE, demonstrated that the UC-to-UU substitutions were highly detectable in nonduplex regions of the vRNA, typically hairpin, bulge or single-stranded regions (Supplementary Figure S3A and B). These nucleotide preferences suggested that host cytidine deaminases specific for single-stranded polynucleotides are continuously involved in the generation of UC-to-UU mutations in the SARS-CoV-2 genome.

Interestingly, in the viral protein-coding regions, the prevalence of synonymous UC-to-UU mutations per site was significantly higher than that of nonsynonymous UC-to-UU mutations (Supplementary Figure S3C), suggesting that the number of SARS-CoV-2 genome mutations driven by host factors and/or viral replication errors might be underestimated in sequence datasets. This underestimation is most likely attributable to certain selective pressures. Importantly, comparative analysis of the substitution frequencies in trinucleotide motifs indicated that the numbers of UUC-to-UUU and ACC-to-ACU mutations were significantly higher than those of mutations in other motifs (Figure 1C). These mutation signatures were consistent with the preferential sequence motifs of APOBEC-mediated deamination and different from RNA polymerization error patterns induced by nsp12s in the related coronaviruses, mouse hepatitis virus (MHV) (43,44) and SARS-CoV-1 (43,45).

### Impacts of exogenous cytidine deaminase expression on the viral genome

**Figure 2.**
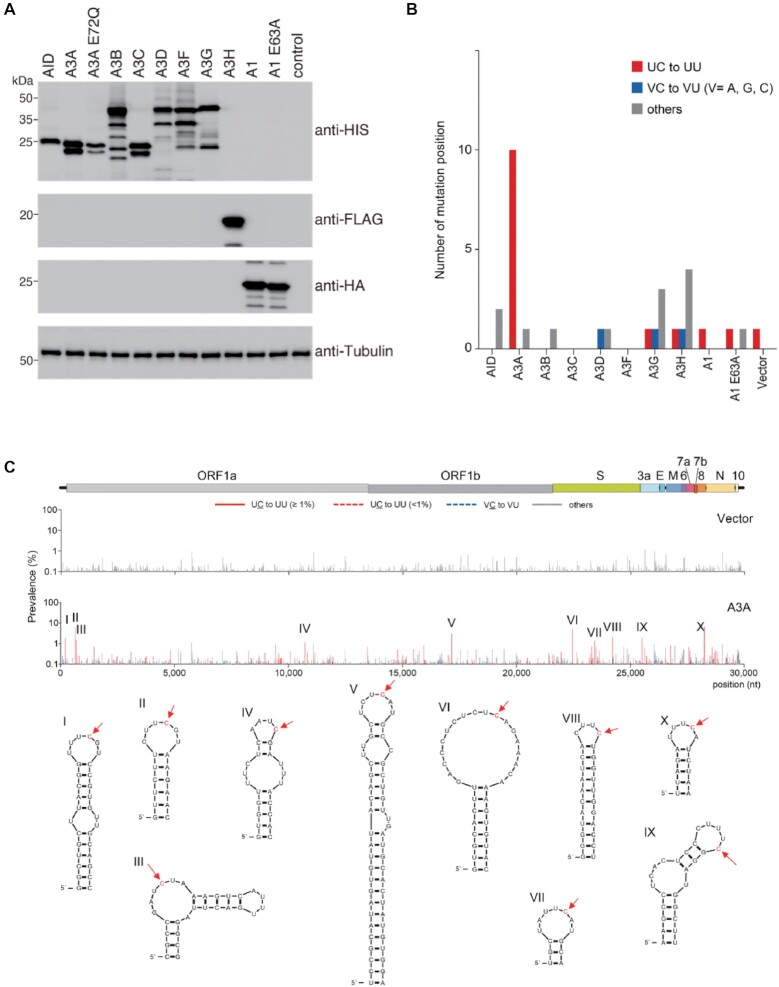
Exogenous A3A expression induces C-to-U mutations in the SARS-CoV-2 vRNA genome, preferentially in ssRNA regions. (**A**) The expression levels of human AID, A3 (A-G, or H haplotype II [hapII]), A1 and A1 E63A proteins in the transfected 293AT cells were analysed by immunoblotting with anti-HIS, anti-FLAG, or anti-HA mAbs. Empty vector (Vector) was used as the negative control for the absence of deaminase expression. An anti-ß-tubulin antibody was used as the loading control. (**B**) The number of positions with C-to-U substitutions in the viral genome was determined. AID, A3 and A1 proteins were transiently expressed in 293AT cells. Thirty-six hours after transfection, the cells were infected with SARS-CoV-2 (B.1.1) (MOI = 0.5) and incubated for another 24 h. Viral genomes were sequenced on the Illumina MiSeq system. The graph shows the number of positions with C-to-U mutations in the dinucleotide context (UC-to-UU, VC-to-VU (V = not U), and others), with a prevalence of ≥ 1.0% throughout the viral genome. The data are representative of three independent experiments. (**C**) The prevalence of mutations detected at each position in the viral genome is indicated in two bar graphs, along with a schematic diagram of the viral genomic structure. The upper and lower graphs represent the mutation prevalence (%) in the viruses produced from 293AT cells transfected with empty vector (Vector) or the A3A plasmid (A3A), respectively. The ten major positions of A3A-induced editing are labelled I-X. Secondary RNA structures around the edited positions (red arrows) were extracted from the results of a previous SHAPE study (40) and are drawn in this figure.

To identify the APOBEC family member(s) that potentially drive viral C-to-U mutations, we performed an in vitro mutation prevalence analysis of the vRNA genome sequences of viruses produced from 293T cells stably expressing human ACE2 and TMPRSS2 (293AT cells). The cells were transiently transfected with DNA encoding AID, A1, or one of the seven human A3s. The amount of each epitope-tagged AID, A3 or A1 in the 293AT cells was confirmed by immunoblot analysis (Figure 2A). Twenty-four hours after infection of the transfected 293AT cells, the culture supernatant was subjected to NGS analysis with the Illumina MiSeq system to identify mutations at each viral genomic position. Mutations found in ≥1% of the population at each position were scored to distinguish minority mutations from sequencing errors (32). Ten positions with UC-to-UU mutation were detected in the vRNA genome of SARS-CoV-2 produced in 293AT cells expressing A3A but not in the genome of SARS-CoV-2 produced in cells expressing any other APOBEC protein (Figure 2B). Expression of the catalytically inactive A3A mutant (A3A E72Q) induced a minimum number of UC-to-UU mutations (Supplementary Figures S4A and B). Interestingly, these A3A-mediated mutations were also observed in other SARS-CoV-2 lineages, namely, the VOCs Alpha, Beta and Gamma (Supplementary Figure S4). These results demonstrated that exogenous expression of A3A but not of the other APOBEC family members increases UC-to-UU mutations in the SARS-CoV-2 RNA genome in vitro. Furthermore, we tested the effects of A3A on the mutation prevalence in the SARS-CoV-2 genome with different expression levels of A3A or A3A E72Q. The results showed that the UC-to-UU mutation prevalence was increased depending on expression levels of wild-type (WT) A3A, but not on those of A3A E72Q (Supplementary Figure S5). The viral titers were similar between the presence and absence of A3A, suggesting that A3A exerts no strong inhibitory effect on SARS-CoV-2 replication cycle.

We mapped the mutation frequencies per site to the SARS-CoV-2 reference sequence and identified 10 loci (I-X) with a high frequency of UC-to-UU mutations (Figure 2C). These loci were located at dispersed positions throughout the entire genomic sequence. Importantly, the edited C bases were located on the 3′ side in middle positions of bulge or loop regions in the vRNA secondary structure (Figure 2C). Moreover, most substitutions in the VOCs Alpha, Beta and Gamma were detected predominantly at these same genomic positions (Supplementary Figure S4C); however, other UC-to-UU mutations with low prevalence were mapped to hairpin loop regions that differed among the lineages. These results indicated that A3A-mediated substitutions in the SARS-CoV-2 RNA genome are strand- and sequence motif-specific and occur preferentially at the tips in loop and bulge regions in the vRNA secondary structure. Notably, these features of A3A-mediated substitutions in the SARS-CoV-2 genome appeared similar to those of cellular RNA editing by A3A in macrophages (18) and DNA oligonucleotide deamination by A3A *in vitro* (46). Moreover, A3A-mediated mutations in the SARS-CoV-2 genome are most likely conserved among *Catarrhini* (primates), because human, chimpanzee, cynomolgus macaque and rhesus macaque A3A orthologues induced UC-to-UU substitutions at similar loci in the viral genome (Supplementary Figure S6). The mutation prevalence displays higher in the presence of human and chimpanzee A3As than the macaque A3As, likely because rhesus A3A has lower catalytic activity than human A3A (47).

### A3A expression in respiratory epithelial cells

**Figure 3.**
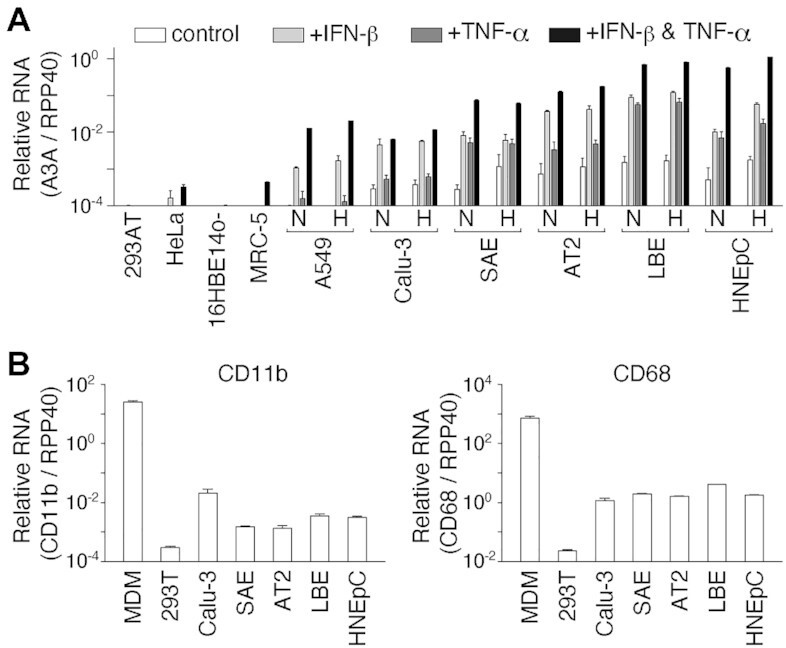
A3A mRNA expression in human airway and lung cells. (**A**) The A3A mRNA level in each cell line was quantified by RT–ddPCR 18 h after treatment without (control) or with IFN-ß and/or TNF-α under normoxic conditions (N). A549, Calu-3, SAE, AT2 and LBE cells and HNEpCs were also cultured under hypoxic conditions (H). The mean mRNA copy numbers of A3A relative to those of the housekeeping gene RPP40 are shown (*n* = 3). (**B**) The CD11b and CD68 mRNA levels in each untreated cell line were quantified by RT–ddPCR. Human MDMs were used as positive controls. The mean mRNA copy numbers of CD11b and CD68 relative to those of RPP40 are shown (*n* = 3). The error bars indicate the + SD values.

Since it is unknown whether A3A is expressed in epithelial cells in the airway and lung tissues, which are primary targets for SARS-CoV-2 infection, we performed quantitative RT–ddPCR to assess the expression of A3A mRNA in cell lines and primary cells of the airway and lung. As shown in Figure 3A, constitutive (control) and IFN-ß-induced expression (+IFN-ß) of A3A mRNA were detected in a cell line-dependent manner, i.e. negligible A3A mRNA expression was observed in 16HBE14o- (a human bronchial epithelial cell line) and MRC-5 (a human foetal lung fibroblast line) cells, whereas high A3A mRNA expression was found in the human lung adenocarcinoma-derived (epithelial-like) cell lines A549 and Calu-3. Moreover, TNF-α had an additive effect on IFN-ß-induced A3A mRNA expression in A549 and Calu-3 cells. In contrast, A3A was constitutively expressed and was excessively expressed after induction by IFN-ß alone or IFN-ß + TNF-α in all primary cells: primary SAE cells showed increases of 29- and 267-fold; human AT2 cells showed increases of 51- and 177-fold; primary human LBE cells showed increases of 59- and 465-fold; and HNEpCs showed increases of 20- and 1,120-fold, by IFN-ß alone and IFN-ß + TNF-α, respectively. Hypoxia treatment (1% oxygen for 18 h) slightly increased IFN-ß-induced A3A expression in primary cells, LBE cells (1.4-fold) and HNEpCs (5.7-fold). Treatment with type II IFN or type III IFNs also induced A3A expression in Calu-3 cells and HNEpCs (Supplementary Figure S7). In contrast, the mRNA expression levels of CD11b and CD68, which are expressed primarily in myeloid lineage cells, such as macrophages, were comparable between Calu-3 cells and primary epithelial cells (SAEs, AT2 cells, LBE cells and HNEpCs) (Figure 3B). This result suggested that the primary epithelial cells used in this study were not significantly contaminated by macrophages, which are abundant in the airways and lungs, as a potential source of the A3A gene.

The A3A protein level was correlated with the A3A mRNA expression level in HNEpCs (Supplementary Figure S7). (Notably, we did not compare A3A protein and mRNA expression in Calu-3 cells because the available anti-A3A antibodies failed to generate sufficient titres for detection by immunoblotting). These results demonstrated that IFN-inducible A3A is expressed in airway and lung epithelial cells, where SARS-CoV-2 replicates efficiently. Interestingly, PMA treatment induced a drastic increase in A3A mRNA expression (∼1.8 × 10^6^-fold relative to control) in HNEpCs, similar to the previously observed effect of phorbolin-1 on keratinocytes (11).

### Endogenous A3A expression and viral mutation signatures

**Figure 4.**
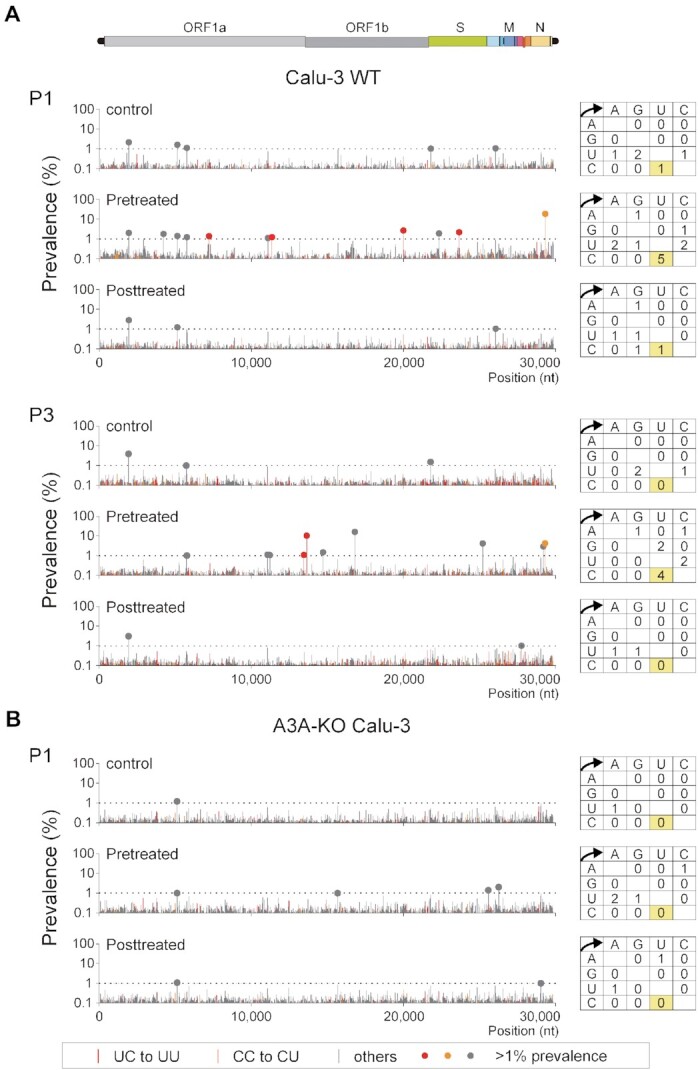
A3A expression induced by IFN-ß and TNF-α in Calu-3 cells increases UC-to-UU mutations in the SARS-CoV-2 genome. (**A** and **B**) Effects of IFN-ß and TNF-α treatment on C-to-U mutations in the SARS-CoV-2 genome were analysed using Calu-3 cells and A3A-KO Calu-3 cells (clone #15). The cells were left untreated (control) or pretreated (Pretreated) with IFN-ß and TNF-α. Seventy-two hours post-infection with SARS-CoV-2 (B.1.1), the culture supernatants were harvested. The infected cells were treated with IFN-ß and TNF-α (Posttreated), and the supernatants were collected. Each harvested virus was further passaged twice in Calu-3 WT cells with the corresponding IFN-ß and TNF-α treatment (A). The position and prevalence of the mutations in the genome at passages 1 (P1) and 3 (P3) were analysed and are shown in bar graphs. The closed circles indicate mutations with a prevalence of ≥1%.

To validate A3A-mediated vRNA editing in virus-targeted cells, we analysed the C-to-U substitution rates in vRNA of SARS-CoV-2 produced in lung-derived epithelial cells with or without IFN-ß and TNF-α stimulation. Because primary cells were less sensitive than Calu-3 cells to SARS-CoV-2 (the B.1.1 lineage strain) in our infection experiments, Calu-3 cells were used for vRNA measurement. Calu-3 cells were infected with virus and were left untreated (Figure 4A, control) or treated with IFN-ß and TNF-α before (Figure 4A, Pretreated) or after (Figure 4A, Posttreated) infection. SARS-CoV-2 is highly sensitive to IFN pretreatment (37–39), leading to an insufficient virus yield for adequate genome coverage at the proper sequencing depth for NGS. Moreover, SARS-CoV-2 antagonizes IFN signalling by expressing viral products such as ORF6 (39,48) (as previously reviewed (49)), which may interfere with IFN-stimulated A3A induction. Therefore, we purposely set a time interval between IFN-ß treatment and virus inoculation to allow stable accumulation of the A3A enzyme in cells. In addition, the harvested virus (P1) was serially passaged two times (P3) following the same treatment strategy. Mutations in P1 and P3 samples were analysed by NGS, scored, and mapped to the viral genome. UC-to-UU substitutions (prevalence of ≥1%) were detected only in the pretreated samples (Figure 4A). Additionally, the substitutions detected in P1 differed from those detected in P3, suggesting that mutations are sporadically induced and selected during virus passaging. Because minor mutations other than UC-to-UU substitutions (prevalence of ≥1%) were also detected in the pretreated P1 and P3 samples (for example, A-to-C, G-to-U and A-to-G mutations at nt positions 11 075, 11 219, 14 712 and 16 807, respectively, in P3) (Figure 4A), treatment with IFN-ß and TNF-α might slightly increase the rates of other substitution patterns.

Finally, to clarify whether the observed UC-to-UU substitutions were induced by A3A in Calu-3 cells, we analysed viral genome mutations in SARS-CoV-2 produced in A3A-knockout (KO) Calu-3 cells treated with or without IFN-ß and TNF-α. A3A-KO Calu-3 cells were generated by the CRISPR/Cas9 system targeting the A3A exon 2 region (Supplementary Figure S8). In two A3A-KO cell clones, #15 and #24, carrying A3A alleles with 37-bp and 76-bp deletions, A3A mRNA expression was abolished in the presence of IFN-ß and TNF-α (Supplementary Figure S8C). Then, A3A-KO Calu-3 (clone #15) cells were infected with SARS-CoV-2 and were left untreated (control) or treated with IFN-ß and TNF-α before or after infection, similar to the treatment scheme for Calu-3 WT cells. Mutations in viral genomes in the supernatants were analysed by NGS, and no significant UC-to-UU substitutions (prevalence of ≥ 1%) were observed in the pretreated sample of A3A-KO Calu-3 cells (Figure 4B). In contrast, two non-C-to-U mutations were detected in the pretreated P1 sample of A3A-KO Calu-3 cells: these mutations may be induced independent of A3A in the presence of IFN-ß and TNF-α. These data suggested that the IFN-ß- and TNF-α-induced upregulation of A3A expression was causally connected to the elevation in the UC-to-UU mutation signature of epithelial cells.

## DISCUSSION

In this study, we demonstrated that expression of exogenous A3A but not other APOBEC enzymes induced UC-to-UU mutations in the SARS-CoV-2 vRNA genome in vitro (Figure 2). These mutation signatures mediated by A3A expression, such as the seven major mutation sites (I-III and VII-X) (Figure 2C), were also observed in multiple lineages in the GISAID data. In addition, we found that upregulation of endogenous A3A expression led to an increase in UC-to-UU mutations in the viral genome in SARS-CoV-2 produced from Calu-3 cells pretreated with IFN-ß and TNF-α (Figure 4). CRISPR/Cas9-mediated A3A KO in Calu-3 cells abrogated the increase in these viral UC-to-UU mutations. This evidence strongly supports the hypothesis that A3A-mediated vRNA editing is a primary mechanism of SARS-CoV-2 mutation by a host factor. However, the mutation positions frequently observed in viral genomes were not completely consistent between the epidemiological datasets and our in vitro experimental data based on exogenous A3A expression (i.e. we found that three major sites, IV-VI, were different between these data sources). In addition, the UC-to-UU mutation rate in the spike (S) region was relatively low according to an epidemiological database but high in our in vitro experimental data (Figure 2C). These discrepancies are likely because the S mutation signatures found in the datasets result from strong immune selection pressure (3). Importantly, expression of exogenous A3A yielded no significant impact on the viral titer (Supplementary Figure S5D), suggesting that A3A does not contribute to restricting the SARS-CoV-2 replication cycle for a short period. In contrast, the results of our epidemiological analysis showed that the UC-to-UU mutations that are preferentially mediated by A3A chronologically continue to occur in the SRAS-CoV-2 genome (Figure 1). Therefore, A3A appears to play a critical role to increase genetic variations in the SARS-CoV-2 genome for a long period, possibly under neutral viral evolution.

Recently, during the preparation of our manuscript, Kim et al. reported the involvement of vRNA editing mediated by A1, A3A and A3G in the occurrence of biased C-to-U substitutions (50), which differs from our results herein. This discrepancy may be largely due to the different *in vitro* assay systems used. Kim et al. coexpressed each of seven 200-nt SARS-CoV-2 RNA fragments with A1, A3A, or A3G in 293T cells by DNA transfection and analysed the substitution rates by NGS. In contrast, we infected 293AT cells transiently expressing APOBEC proteins with live SARS-CoV-2 and analysed the mutation rates of the whole viral genome by NGS (Figure 2). In cells, SARS-CoV-2 vRNA is localized predominantly in the cytoplasm where replication occurs; however, RNA fragments expressed by transfection are generally localized in both the nucleus and cytoplasm. Since APOBEC-mediated deamination events should depend on the specific localization of each enzyme in cells, assessments of RNA editing in SARS-CoV-2 genome fragments expressed by transfection may produce misleading results. For example, A1/APOBEC1-complementation factor (A1CF)-mediated deamination occurs predominantly in the nucleus (51), where SARS-CoV-2 vRNA is not present. Additionally, whether A1 and A3G are physiologically involved in vRNA substitutions remains unclear, because the A1 and A3G enzymes are expressed predominantly in intestinal cells (20,52) and lymphoid/myeloid cells (15), respectively, which are not primary targets for SARS-CoV-2 replication and transmission. Therefore, the possibility that both A1 and A3G are involved in driving U-to-C mutations in the SARS-CoV-2 RNA genome under physiological and virological conditions appears unconvincing. Furthermore, although previous reports have shown that A3G deaminates cellular RNAs in macrophages (23), whether A3G catalyses the deamination of RNA substrates remains controversial for two reasons: 1) previous biochemical analyses using in vitro reaction systems demonstrated that A3G exclusively deaminates cytidines in ssDNA and not in RNA substrates (53–55), and 2) accumulated studies on A3G-mediated antiretroviral activity have shown that A3G incorporated in retroviral particles does not exhibit deamination activity targeting cytosines in retroviral RNA (10). Further investigations on RNA editing events mediated by A3G in cells are required.

Another deaminase, A3B, which induces mutational signatures similar to those induced by A3A in the chromosomal DNA genome (56), exerted no significant effect on the C-to-U substitution rate in the SARS-CoV-2 genome (Figure 2). The in vitro data was consistent with the epidemiological evidence showing that the chronological C-to-U mutation rates were equivalent between sequences of SARS-CoV-2 isolated in different global regions, including Oceania and the Malay Archipelago (Supplementary Figure S1), which exhibit high frequencies of the *A3B* gene deletion polymorphism (57). Additionally, A3B localizes predominantly to the nucleus (58), which is not the site of SARS-CoV-2 replication.

However, a question has been raised about how A3A efficiently deaminates both RNA and ssDNA but A3G and A3B exclusively deaminate ssDNA despite the phylogenetic similarity of the catalytic domains in the A3G and A3B C-terminal domains (CTDs) to A3A as the same Z1 type (10). Structural information might provide important insight to answer this question. Our results (Figure 2C) and the results of other previous studies have shown that A3A preferentially deaminates cytosines in hairpin sequences of ssDNA (56,59) and ssRNA (18). Based on the molecular structures of the A3A-ssDNA complex (Protein Data Bank ID 5SWW) (59), the ssDNA substrate containing three nucleotides – i.e. the deamination target deoxycytidine (dC_0_) and the flanking deoxycytidines (dT_-1_ and dT_-2_) – are docked in the substrate cavity of A3A (60). The trinucleotide recognized by A3A adopts the C2′-endo sugar pucker conformation at dC_0_ and dT_-1_. In general, ssRNA is predominantly in the C3′-endo conformation, although the riboses at the tips of RNA hairpins tend to adopt the C2′-endo sugar pucker conformation (61). Therefore, A3A preferentially recognizes ssDNA as well as RNA hairpins (with a nonhelical C2′-endo sugar pucker conformation similar to ssDNA), as supported by our structural modelling analysis (Supplementary Figure S9A). However, the CTDs of both A3B and A3G have bulky loop 1 regions that protrude into the substrate binding pockets and prevent the accommodation of hairpin RNAs (Supplementary Figure S9). Structural determinations of the A3A-hairpin RNA complex are warranted to indicate the mechanistic hypothesis that among the A3 family enzymes, only A3A efficiently deaminates both ssDNA and RNA.

In SARS-CoV-2-infected cells, vRNAs, including negative-strand vRNA (–vRNA) intermediates, are synthesized in an occluded cellular compartment called a double-membrane vesicle (DMV), which prevents the detection of vRNA intermediates by innate immune sensors (62,63). Since A3A appears to have difficulty targeting –vRNA in DMVs for deamination, the strand-biased UC-to-UU substitutions mediated by A3A are the most likely to be detected in the SARS-CoV-2 genome. SARS-CoV-2 replication is largely divided into two phases, post-virus entry through DMV compartmentalization (the early phase) and virus assembly (the late phase), in which A3A effectively targets the vRNA genome in the cytoplasm. As shown in Figure 4, IFN-ß and TNF-α treatment before SARS-CoV-2 infection – but not after infection – increased the number of C-to-U substitutions in the vRNA genome in Calu-3 cells. This early-phase dependency might be explained by two mechanisms. (i) SARS-CoV-2 may be more sensitive to A3A-mediated editing in the early phase of virus replication, or (ii) A3A may not be sufficiently induced by IFN-ß because certain viral gene products, such as ORF6, suppress IFN signalling (39,48), which is required for A3A induction. To date, we have not identified A3A inducers (except for the combination of IFN-ß and TNF-α) that are truly active in COVID-19 patients in vivo. Since PMA induced a drastic increase in the A3A level in primary nasal epithelial cells (Supplementary Figure S7), PMA-related signalling pathways might be key for clarifying A3A regulation in bronchial tissue epithelial cells.

Overall, our study provides multiple insights into genetic changes in SARS-CoV-2 mediated in association with host factors. To date, cytidine deamination of DNA by A3A has been extensively studied in terms of antiviral (mainly antiretroviral) activity. All these findings suggest new possibilities for understanding the effects of A3A on the genetic diversification of RNA viruses, such as SARS-CoV-2 and other *Coronaviridae* family members that replicate efficiently in the respiratory epithelium.

## DATA AVAILABILITY

The SARS-CoV-2 pangenomic sequence data used in this study are available at the GISAID EpiCoV portal (https://www.gisaid.org/). The deep sequencing data obtained in this study have been deposited in the DNA Data Bank of Japan (DDBJ) under accession ID PRJDB13356.

## Supplementary Material

gkac1238_Supplemental_File

## References

[1] Wu F. , Zhao S., Yu B., Chen Y.M., Wang W., Song Z.G., Hu Y., Tao Z.W., Tian J.H., Pei Y.Y. et al. A new coronavirus associated with human respiratory disease in China. Nature. 2020; 579:265–269. 32015508 10.1038/s41586-020-2008-3 PMC7094943

[2] Zhu N. , Zhang D., Wang W., Li X., Yang B., Song J., Zhao X., Huang B., Shi W., Lu R. et al. A novel coronavirus from patients with pneumonia in China, 2019. N. Engl. J. Med. 2020; 382:727–733. 31978945 10.1056/NEJMoa2001017 PMC7092803

[3] Li J. , Lai S., Gao G.F., Shi W. The emergence, genomic diversity and global spread of SARS-CoV-2. Nature. 2021; 600:408–418. 34880490 10.1038/s41586-021-04188-6

[4] Frost S.D.W. , Magalis B.R., Kosakovsky Pond S.L. Neutral theory and rapidly evolving viral pathogens. Mol. Biol. Evol. 2018; 35:1348–1354. 29688481 10.1093/molbev/msy088 PMC6279309

[5] Robson F. , Khan K.S., Le T.K., Paris C., Demirbag S., Barfuss P., Rocchi P., Ng W.L. Coronavirus RNA proofreading: molecular basis and therapeutic targeting. Mol. Cell. 2020; 80:1136–1138. 33338403 10.1016/j.molcel.2020.11.048 PMC7833706

[6] Di Giorgio S. , Martignano F., Torcia M.G., Mattiuz G., Conticello S.G. Evidence for host-dependent RNA editing in the transcriptome of SARS-CoV-2. Sci. Adv. 2020; 6:eabb5813. 32596474 10.1126/sciadv.abb5813 PMC7299625

[7] van Dorp L. , Richard D., Tan C.C.S., Shaw L.P., Acman M., Balloux F. No evidence for increased transmissibility from recurrent mutations in SARS-CoV-2. Nat. Commun. 2020; 11:5986. 33239633 10.1038/s41467-020-19818-2 PMC7688939

[8] Simmonds P. Rampant C→U hypermutation in the genomes of SARS-CoV-2 and other coronaviruses: causes and consequences for their short- and long-term evolutionary trajectories. mSphere. 2020; 5:e00408–e00420. 32581081 10.1128/mSphere.00408-20 PMC7316492

[9] Harris R.S. , Dudley J.P. APOBECs and virus restriction. Virology. 2015; 479-480:131–145. 25818029 10.1016/j.virol.2015.03.012 PMC4424171

[10] Salter J.D. , Bennett R.P., Smith H.C. The APOBEC protein family: united by structure, divergent in function. Trends Biochem. Sci. 2016; 41:578–594. 27283515 10.1016/j.tibs.2016.05.001 PMC4930407

[11] Madsen P. , Anant S., Rasmussen H.H., Gromov P., Vorum H., Dumanski J.P., Tommerup N., Collins J.E., Wright C.L., Dunham I. et al. Psoriasis upregulated phorbolin-1 shares structural but not functional similarity to the mRNA-editing protein apobec-1. J. Invest. Dermatol. 1999; 113:162–169. 10469298 10.1046/j.1523-1747.1999.00682.x

[12] Law E.K. , Levin-Klein R., Jarvis M.C., Kim H., Argyris P.P., Carpenter M.A., Starrett G.J., Temiz N.A., Larson L.K., Durfee C. et al. APOBEC3A catalyzes mutation and drives carcinogenesis in vivo. J. Exp. Med. 2020; 217:e20200261. 32870257 10.1084/jem.20200261 PMC7953736

[13] Burns M.B. , Lackey L., Carpenter M.A., Rathore A., Land A.M., Leonard B., Refsland E.W., Kotandeniya D., Tretyakova N., Nikas J.B. et al. APOBEC3B is an enzymatic source of mutation in breast cancer. Nature. 2013; 494:366–370. 23389445 10.1038/nature11881 PMC3907282

[14] Jalili P. , Bowen D., Langenbucher A., Park S., Aguirre K., Corcoran R.B., Fleischman A.G., Lawrence M.S., Zou L., Buisson R. Quantification of ongoing APOBEC3A activity in tumor cells by monitoring RNA editing at hotspots. Nat. Commun. 2020; 11:2971. 32532990 10.1038/s41467-020-16802-8 PMC7293259

[15] Koning F.A. , Newman E.N., Kim E.Y., Kunstman K.J., Wolinsky S.M., Malim M.H. Defining APOBEC3 expression patterns in human tissues and hematopoietic cell subsets. J. Virol. 2009; 83:9474–9485. 19587057 10.1128/JVI.01089-09 PMC2738220

[16] Refsland E.W. , Stenglein M.D., Shindo K., Albin J.S., Brown W.L., Harris R.S. Quantitative profiling of the full APOBEC3 mRNA repertoire in lymphocytes and tissues: implications for HIV-1 restriction. Nucleic Acids Res. 2010; 38:4274–4284. 20308164 10.1093/nar/gkq174 PMC2910054

[17] Berger G. , Durand S., Fargier G., Nguyen X.N., Cordeil S., Bouaziz S., Muriaux D., Darlix J.L., Cimarelli A. APOBEC3A is a specific inhibitor of the early phases of HIV-1 infection in myeloid cells. PLoS Pathog. 2011; 7:e1002221. 21966267 10.1371/journal.ppat.1002221 PMC3178557

[18] Sharma S. , Patnaik S.K., Taggart R.T., Kannisto E.D., Enriquez S.M., Gollnick P., Baysal B.E. APOBEC3A cytidine deaminase induces RNA editing in monocytes and macrophages. Nat. Commun. 2015; 6:6881. 25898173 10.1038/ncomms7881 PMC4411297

[19] Driscoll D.M. , Lakhe-Reddy S., Oleksa L.M., Martinez D. Induction of RNA editing at heterologous sites by sequences in apolipoprotein B mRNA. Mol. Cell. Biol. 1993; 13:7288–7294. 8246950 10.1128/mcb.13.12.7288-7294.1993 PMC364799

[20] Powell L.M. , Wallis S.C., Pease R.J., Edwards Y.H., Knott T.J., Scott J. A novel form of tissue-specific RNA processing produces apolipoprotein-B48 in intestine. Cell. 1987; 50:831–840. 3621347 10.1016/0092-8674(87)90510-1

[21] Teng B. , Burant C.F., Davidson N.O. Molecular cloning of an apolipoprotein B messenger RNA editing protein. Science. 1993; 260:1816–1819. 8511591 10.1126/science.8511591

[22] Alqassim E.Y. , Sharma S., Khan A., Emmons T.R., Cortes Gomez E., Alahmari A., Singel K.L., Mark J., Davidson B.A., Robert McGray A.J. et al. RNA editing enzyme APOBEC3A promotes pro-inflammatory M1 macrophage polarization. Commun Biol. 2021; 4:102. 33483601 10.1038/s42003-020-01620-x PMC7822933

[23] Sharma S. , Patnaik S.K., Taggart R.T., Baysal B.E. The double-domain cytidine deaminase APOBEC3G is a cellular site-specific RNA editing enzyme. Sci. Rep. 2016; 6:39100. 27974822 10.1038/srep39100 PMC5156925

[24] Matsuoka K. , Imahashi N., Ohno M., Ode H., Nakata Y., Kubota M., Sugimoto A., Imahashi M., Yokomaku Y., Iwatani Y. SARS-CoV-2 accessory protein ORF8 is secreted extracellularly as a glycoprotein homodimer. J. Biol. Chem. 2022; 298:101724. 35157849 10.1016/j.jbc.2022.101724 PMC8832879

[25] Kondo T. , Iwatani Y., Matsuoka K., Fujino T., Umemoto S., Yokomaku Y., Ishizaki K., Kito S., Sezaki T., Hayashi G. et al. Antibody-like proteins that capture and neutralize SARS-CoV-2. Sci. Adv. 2020; 6:eabd3916. 32948512 10.1126/sciadv.abd3916 PMC7556756

[26] Reed L.J. , Muench H. A simple method of estimating fifty percent endpoints. Am. J. Epidemiol. 1938; 27:493–497.

[27] Ikeda T. , Abd El Galil K.H., Tokunaga K., Maeda K., Sata T., Sakaguchi N., Heidmann T., Koito A. Intrinsic restriction activity by apolipoprotein B mRNA editing enzyme APOBEC1 against the mobility of autonomous retrotransposons. Nucleic. Acids. Res. 2011; 39:5538–5554. 21398638 10.1093/nar/gkr124 PMC3141244

[28] Goila-Gaur R. , Khan M.A., Miyagi E., Kao S., Strebel K. Targeting APOBEC3A to the viral nucleoprotein complex confers antiviral activity. Retrovirology. 2007; 4:61. 17727729 10.1186/1742-4690-4-61 PMC2018723

[29] Harari A. , Ooms M., Mulder L.C., Simon V. Polymorphisms and splice variants influence the antiretroviral activity of human APOBEC3H. J. Virol. 2009; 83:295–303. 18945781 10.1128/JVI.01665-08 PMC2612324

[30] Kitamura S. , Ode H., Nakashima M., Imahashi M., Naganawa Y., Kurosawa T., Yokomaku Y., Yamane T., Watanabe N., Suzuki A. et al. The APOBEC3C crystal structure and the interface for HIV-1 Vif binding. Nat. Struct. Mol. Biol. 2012; 19:1005–1010. 23001005 10.1038/nsmb.2378

[31] Imahashi M. , Izumi T., Watanabe D., Imamura J., Matsuoka K., Ode H., Masaoka T., Sato K., Kaneko N., Ichikawa S. et al. Lack of association between intact/deletion polymorphisms of the APOBEC3B gene and HIV-1 risk. PLoS One. 2014; 9:e92861. 24667791 10.1371/journal.pone.0092861 PMC3965477

[32] Ode H. , Matsuda M., Matsuoka K., Hachiya A., Hattori J., Kito Y., Yokomaku Y., Iwatani Y., Sugiura W. Quasispecies analyses of the HIV-1 near-full-length genome with Illumina MiSeq. Front Microbiol. 2015; 6:1258. 26617593 10.3389/fmicb.2015.01258 PMC4641896

[33] Kechin A. , Boyarskikh U., Kel A., Filipenko M. cutPrimers: a new tool for accurate cutting of primers from reads of targeted next generation sequencing. J. Comput. Biol. 2017; 24:1138–1143. 28715235 10.1089/cmb.2017.0096

[34] Li H. , Durbin R. Fast and accurate long-read alignment with Burrows-Wheeler transform. Bioinformatics. 2010; 26:589–595. 20080505 10.1093/bioinformatics/btp698 PMC2828108

[35] 1000 Genome Project Data Processing Subgroup Li H. , Handsaker B., Wysoker A., Fennell T., Ruan J., Homer N., Marth G., Abecasis G., Durbin R. The sequence alignment/map format and samtools. Bioinformatics. 2009; 25:2078–2079. 19505943 10.1093/bioinformatics/btp352 PMC2723002

[36] Kaida A. , Miura M. Differential dependence on oxygen tension during the maturation process between monomeric Kusabira Orange 2 and monomeric Azami Green expressed in HeLa cells. Biochem. Biophys. Res. Commun. 2012; 421:855–859. 22554525 10.1016/j.bbrc.2012.04.102

[37] Katsura H. , Sontake V., Tata A., Kobayashi Y., Edwards C.E., Heaton B.E., Konkimalla A., Asakura T., Mikami Y., Fritch E.J. et al. Human lung stem cell-based alveolospheres provide insights into SARS-CoV-2-mediated interferon responses and pneumocyte dysfunction. Cell Stem Cell. 2020; 27:890–904. 33128895 10.1016/j.stem.2020.10.005 PMC7577733

[38] Lokugamage K.G. , Hage A., de Vries M., Valero-Jimenez A.M., Schindewolf C., Dittmann M., Rajsbaum R., Menachery V.D. Type I interferon susceptibility distinguishes SARS-CoV-2 from SARS-CoV. J. Virol. 2020; 94:e01410–e01420. 10.1128/JVI.01410-20 PMC7654262 32938761

[39] Miorin L. , Kehrer T., Sanchez-Aparicio M.T., Zhang K., Cohen P., Patel R.S., Cupic A., Makio T., Mei M., Moreno E. et al. SARS-CoV-2 Orf6 hijacks Nup98 to block STAT nuclear import and antagonize interferon signaling. Proc. Natl. Acad. Sci. U.S.A. 2020; 117:28344–28354. 33097660 10.1073/pnas.2016650117 PMC7668094

[40] Manfredonia I. , Nithin C., Ponce-Salvatierra A., Ghosh P., Wirecki T.K., Marinus T., Ogando N.S., Snijder E.J., van Hemert M.J., Bujnicki J.M. et al. Genome-wide mapping of SARS-CoV-2 RNA structures identifies therapeutically-relevant elements. Nucleic Acids Res. 2020; 48:12436–12452. 33166999 10.1093/nar/gkaa1053 PMC7736786

[41] Zuker M. Mfold web server for nucleic acid folding and hybridization prediction. Nucleic Acids Res. 2003; 31:3406–3415. 12824337 10.1093/nar/gkg595 PMC169194

[42] Hadfield J. , Megill C., Bell S.M., Huddleston J., Potter B., Callender C., Sagulenko P., Bedford T., Neher R.A. Nextstrain: real-time tracking of pathogen evolution. Bioinformatics. 2018; 34:4121–4123. 29790939 10.1093/bioinformatics/bty407 PMC6247931

[43] Graepel K.W. , Lu X., Case J.B., Sexton N.R., Smith E.C., Denison M.R. Proofreading-deficient coronaviruses adapt for increased fitness over long-term passage without reversion of exoribonuclease-inactivating mutations. mBio. 2017; 8:e01503–e01517. 29114026 10.1128/mBio.01503-17 PMC5676041

[44] Eckerle L.D. , Lu X., Sperry S.M., Choi L., Denison M.R. High fidelity of murine hepatitis virus replication is decreased in nsp14 exoribonuclease mutants. J. Virol. 2007; 81:12135–12144. 17804504 10.1128/JVI.01296-07 PMC2169014

[45] Smith E.C. , Blanc H., Surdel M.C., Vignuzzi M., Denison M.R. Coronaviruses lacking exoribonuclease activity are susceptible to lethal mutagenesis: evidence for proofreading and potential therapeutics. PLoS Pathog. 2013; 9:e1003565. 23966862 10.1371/journal.ppat.1003565 PMC3744431

[46] Buisson R. , Langenbucher A., Bowen D., Kwan E.E., Benes C.H., Zou L., Lawrence M.S. Passenger hotspot mutations in cancer driven by APOBEC3A and mesoscale genomic features. Science. 2019; 364:eaaw2872. 31249028 10.1126/science.aaw2872 PMC6731024

[47] Caval V. , Suspene R., Vartanian J.P., Wain-Hobson S. Orthologous mammalian APOBEC3A cytidine deaminases hypermutate nuclear DNA. Mol. Biol. Evol. 2014; 31:330–340. 24162735 10.1093/molbev/mst195

[48] Schroeder S. , Pott F., Niemeyer D., Veith T., Richter A., Muth D., Goffinet C., Muller M.A., Drosten C. Interferon antagonism by SARS-CoV-2: a functional study using reverse genetics. Lancet Microbe. 2021; 2:e210–e218. 33969329 10.1016/S2666-5247(21)00027-6 PMC8096348

[49] Park A. , Iwasaki A. Type I and type III interferons - induction, signaling, evasion, and application to combat COVID-19. Cell Host Microbe. 2020; 27:870–878. 32464097 10.1016/j.chom.2020.05.008 PMC7255347

[50] Kim K. , Calabrese P., Wang S., Qin C., Rao Y., Feng P., Chen X.S. The roles of APOBEC-mediated RNA editing in SARS-CoV-2 mutations, replication and fitness. Sci. Rep. 2022; 12:14972. 36100631 10.1038/s41598-022-19067-x PMC9470679

[51] Lehmann D.M. , Galloway C.A., Sowden M.P., Smith H.C. Metabolic regulation of apoB mRNA editing is associated with phosphorylation of APOBEC-1 complementation factor. Nucleic Acids Res. 2006; 34:3299–3308. 16820530 10.1093/nar/gkl417 PMC1500872

[52] Blanc V. , Xie Y., Kennedy S., Riordan J.D., Rubin D.C., Madison B.B., Mills J.C., Nadeau J.H., Davidson N.O. Apobec1 complementation factor (A1CF) and RBM47 interact in tissue-specific regulation of C to U RNA editing in mouse intestine and liver. RNA. 2019; 25:70–81. 30309881 10.1261/rna.068395.118 PMC6298562

[53] Iwatani Y. , Takeuchi H., Strebel K., Levin J.G. Biochemical activities of highly purified, catalytically active human APOBEC3G: correlation with antiviral effect. J. Virol. 2006; 80:5992–6002. 16731938 10.1128/JVI.02680-05 PMC1472592

[54] Solomon W.C. , Myint W., Hou S., Kanai T., Tripathi R., Kurt Yilmaz N., Schiffer C.A., Matsuo H. Mechanism for APOBEC3G catalytic exclusion of RNA and non-substrate DNA. Nucleic Acids Res. 2019; 47:7676–7689. 31424549 10.1093/nar/gkz550 PMC6698744

[55] Suspene R. , Sommer P., Henry M., Ferris S., Guetard D., Pochet S., Chester A., Navaratnam N., Wain-Hobson S., Vartanian J.P. APOBEC3G is a single-stranded DNA cytidine deaminase and functions independently of HIV reverse transcriptase. Nucleic Acids Res. 2004; 32:2421–2429. 15121899 10.1093/nar/gkh554 PMC419444

[56] Langenbucher A. , Bowen D., Sakhtemani R., Bournique E., Wise J.F., Zou L., Bhagwat A.S., Buisson R., Lawrence M.S. An extended APOBEC3A mutation signature in cancer. Nat. Commun. 2021; 12:1602. 33707442 10.1038/s41467-021-21891-0 PMC7952602

[57] Kidd J.M. , Newman T.L., Tuzun E., Kaul R., Eichler E.E. Population stratification of a common APOBEC gene deletion polymorphism. PLoS Genet. 2007; 3:e63. 17447845 10.1371/journal.pgen.0030063 PMC1853121

[58] Lackey L. , Demorest Z.L., Land A.M., Hultquist J.F., Brown W.L., Harris R.S. APOBEC3B and AID have similar nuclear import mechanisms. J. Mol. Biol. 2012; 419:301–314. 22446380 10.1016/j.jmb.2012.03.011 PMC3368237

[59] Shi K. , Carpenter M.A., Banerjee S., Shaban N.M., Kurahashi K., Salamango D.J., McCann J.L., Starrett G.J., Duffy J.V., Demir O. et al. Structural basis for targeted DNA cytosine deamination and mutagenesis by APOBEC3A and APOBEC3B. Nat. Struct. Mol. Biol. 2017; 24:131–139. 27991903 10.1038/nsmb.3344 PMC5296220

[60] Hou S. , Lee J.M., Myint W., Matsuo H., Kurt Yilmaz N., Schiffer C.A. Structural basis of substrate specificity in human cytidine deaminase family APOBEC3s. J. Biol. Chem. 2021; 297:100909. 34171358 10.1016/j.jbc.2021.100909 PMC8313598

[61] Puglisi J.D. , Wyatt J.R., Tinoco I. Jr. Solution conformation of an RNA hairpin loop. Biochemistry. 1990; 29:4215–4226. 1694459 10.1021/bi00469a026

[62] Snijder E.J. , Limpens R., de Wilde A.H., de Jong A.W.M., Zevenhoven-Dobbe J.C., Maier H.J., Faas F., Koster A.J., Barcena M. A unifying structural and functional model of the coronavirus replication organelle: tracking down RNA synthesis. PLoS Biol. 2020; 18:e3000715. 32511245 10.1371/journal.pbio.3000715 PMC7302735

[63] Wolff G. , Limpens R., Zevenhoven-Dobbe J.C., Laugks U., Zheng S., de Jong A.W.M., Koning R.I., Agard D.A., Grunewald K., Koster A.J. et al. A molecular pore spans the double membrane of the coronavirus replication organelle. Science. 2020; 369:1395–1398. 32763915 10.1126/science.abd3629 PMC7665310

