# Supplementary material for: Cellular APOBEC3A deaminase drives mutations in the SARS-CoV-2 genome
Source: Nucleic Acids Res. 2023 Jan 5;51(2):783–95. doi: 10.1093/nar/gkac1238 (PMC9881129; doi:10.1093/nar/gkac1238)
Supplement: gkac1238_Supplemental_File [file gkac1238_supplemental_file.pdf]

Supplementary Data

Cellular APOBEC3A deaminase drives mutations in the SARS-CoV-2 genome

Yoshihiro Nakata, Hirotaka Ode, Mai Kubota, Takaaki Kasahara, Kazuhiro Matsuoka, Atsuko Sugimoto, Mayumi Imahashi, Yoshiyuki Yokomaku, & Yasumasa Iwatani

Overview

Supplementary Figure S1: Similar chronological nucleotide changes in SARS-CoV-2

Supplementary Figure S2: Chronological dinucleotide changes in SARS-CoV-2 lineages

Supplementary Figure S3: UC-to-UU mutation signatures within the SARS-CoV-2 genome

Supplementary Figure S4: Effects of A3A deaminase activity on UC-to-UU mutations

Supplementary Figure S5: Effects of different A3A expression levels on UC-to-UU mutations and viral titer

Supplementary Figure S6: Effects of A3A deaminase activity on UC-to-UU mutations

Supplementary Figure S7: A3A induction by IFNs and proinflammatory cytokines in cells

Supplementary Figure S8: Validation of A3A-KO Calu-3 cells generated by CRISPR/Cas9

Supplementary Figure S9: Structural diagram of A3A-mediated RNA editing

Supplementary Table S1: cDNA sequences of primate A3A orthologues

Supplementary Table S2: Oligonucleotides used in this study

Supplementary Methods: Brief description of the methods for the Supplementary Figures

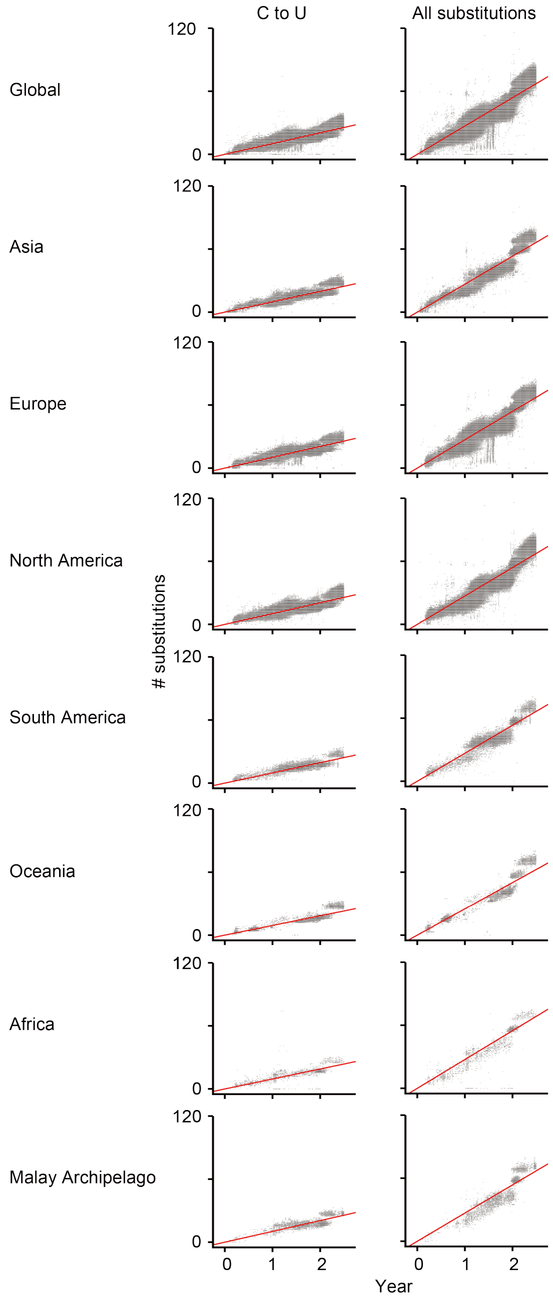

Supplementary Figure S1. Similar chronological nucleotide changes in SARS-CoV-2 are observed in different geographical regions. Chronological changes in the number of substitutions were analysed in viruses sampled in all regions (Global), Asia, Europe, North America, South America, Oceania, Africa, and the Malay Archipelago. The numbers of C-to-U mutations and all other mononucleotide mutations within the SARS-CoV-2 genome sequences (n = 2,051,393) were plotted against the sample collection date based on the information in the GISAID database. The numbers of mutations in the SARS-CoV-2 samples collected in the Malay Archipelago were analysed separately from those in Asia because the *A3B* gene deletion polymorphism is highly prevalent in the Malay region (Brunei, East Timor, Indonesia, Malaysia, Papua New Guinea, the Philippines, and Singapore). The years “0”, “1” and “2” denote 2020, 2021 and 2022, respectively. The red lines represent linear regression lines calculated by fitting the input data to a linear function (y = a*x). The graphs were generated with GNUPLOT ver. 5.2 (http://www.gnuplot.info/).

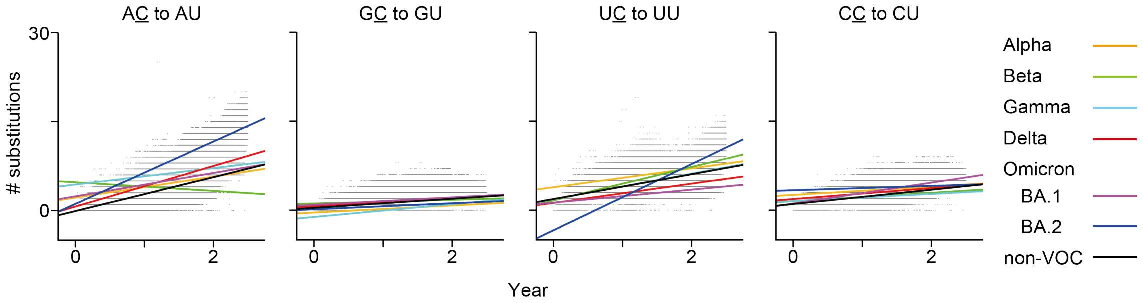

Supplementary Figure S2. Chronological UC-to-UU dinucleotide changes in the SARS-CoV-2 genome are independent of viral lineage. The number of C-to-U substitutions in the dinucleotide context (AC, GC, UC and CC) was plotted (grey dots) against the sample collection date. Linear regression lines for each data point for the Alpha (orange), Beta (green), Gamma (cyan), Delta (red), Omicron BA.1 (purple), Omicron BA.2 (blue) variants and non-VOC lineages (black) were calculated by fitting the input data to a linear function (y = a*x + b). The graphs were generated with GNUPLOT ver. 5.2 (http://www.gnuplot.info/). The years “0” and “2” denote 2020 and 2022, respectively. Both the AC-to-AU and UC-to-UU mutations chronologically accumulated at higher rates than the other two patterns (GC-to-GU and CC-to-CU), although changes in UC-to-UU mutations but not AC-to-AU mutations were observed in viral lineages.

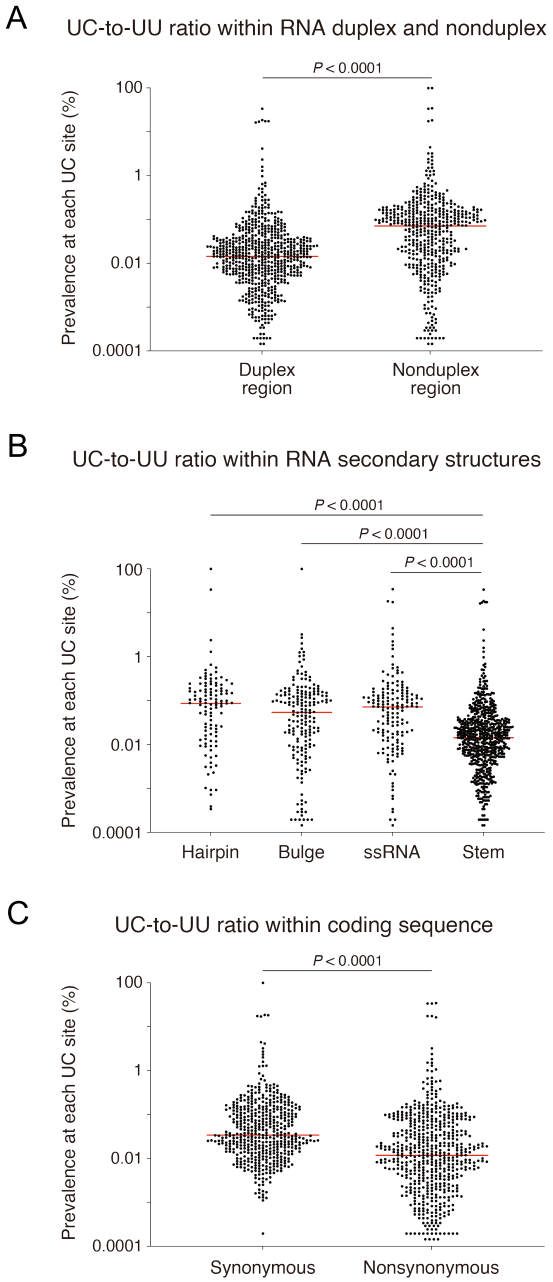

Supplementary Figure S3. The UC-to-UU mutation signatures within the SARS-CoV-2 genome sequences in the GISAID database show genetic bias. (**A** and **B**) The prevalence of UC-to-UU substitutions in whole-genome sequences (n = 2,051,393) was compared at each site in the secondary RNA structures, as indicated by the SHAPE data in a previous report: (**A**) between RNA duplex (635 sites) and nonduplex (472 sites) regions, and (**B**) in hairpin (119 sites), bulge (191 sites), other ssRNA regions (162 sites), and stem regions (635 sites). (**C**) The prevalence of synonymous (487 sites) and nonsynonymous (589 sites) UC-to-UU mutations at each UC dinucleotide within the protein-coding region of the genome was compared. Mutations with a prevalence of less than 0.0001% are not shown in the graphs. The red line indicates the median. Significance was analysed by the Mann–Whitney U test (**A** and **C**) or by a post hoc multiple comparison test (**B**).

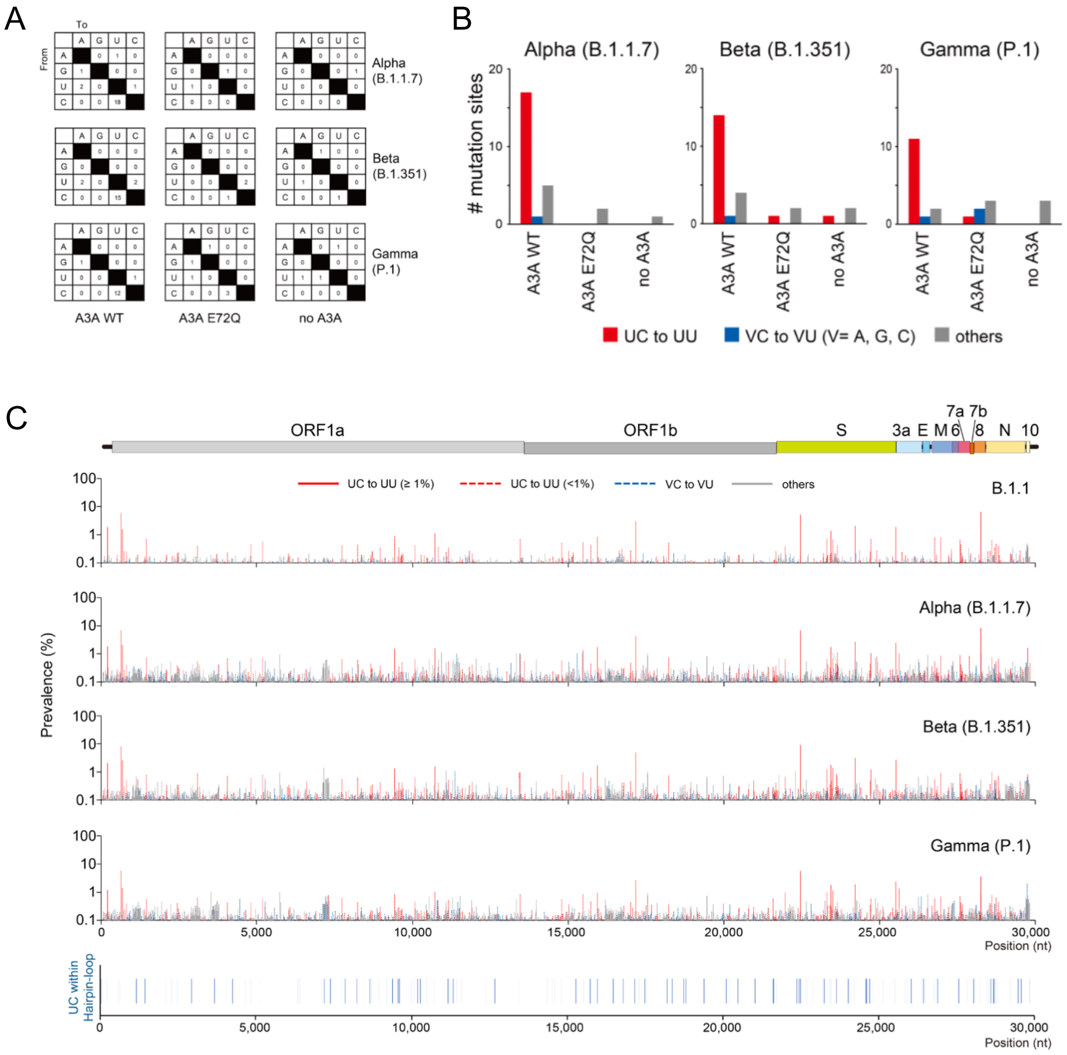

Supplementary Figure S4. A3A deaminase activity is required for the induction of genomic UC-to-UU mutations in three SARS-CoV-2 VOCs. (**A** and **B**) Mononucleotide substitutions in the genomes of the VOCs were analysed using 293AT cells in which wild-type (WT) A3A or the deaminase-deficient A3A mutant E72Q was transiently expressed via transfection. Empty vector was used as the negative control (no A3A). Thirty-six hours after transfection, 293AT cells were infected with a VOC (MOI = 0.5) –Alpha (B.1.1.7), Beta (B.1.351) or Gamma (P.1) (Supplementary Methods). Twenty-four hours post-infection, cell culture supernatants were harvested. (**A**) Mutation matrices showing substitution positions with a prevalence of ≥1% in each sample. (**B**) The prevalence of C-to-U mutations at different dinucleotide motifs is shown. The numbers of genomic positions where the prevalence of U<u>C</u>-to-UU, V<u>C</u>-to-VU (V = not U), and other C-to-U mutations was equal to or greater than 1% are shown. (**C**) The prevalence of mutations detected at each viral genomic position is shown in bar graphs, along with a schematic diagram of the viral genomic structure. 293AT cells transfected with the human A3A expression plasmid were infected with SARS-CoV-2 B.1.1, Alpha, Beta, or Gamma. The culture supernatants were used for viral genome analysis by deep sequencing. The prevalence (%) of U<u>C</u>-to-UU mutations (red lines), V<u>C</u>-to-VU mutations (blue dotted line), and other mutations (grey line) at each position in the SARS-CoV-2 genome are shown. The solid and dotted red lines indicate a prevalence of ≥ 1% and < 1%, respectively. The bottom graph shows the UC dinucleotide sites (blue lines) located in RNA hairpin loop regions of the SARS-CoV-2 Wuhan reference sequence, as indicated by data obtained from a previous SHAPE analysis.

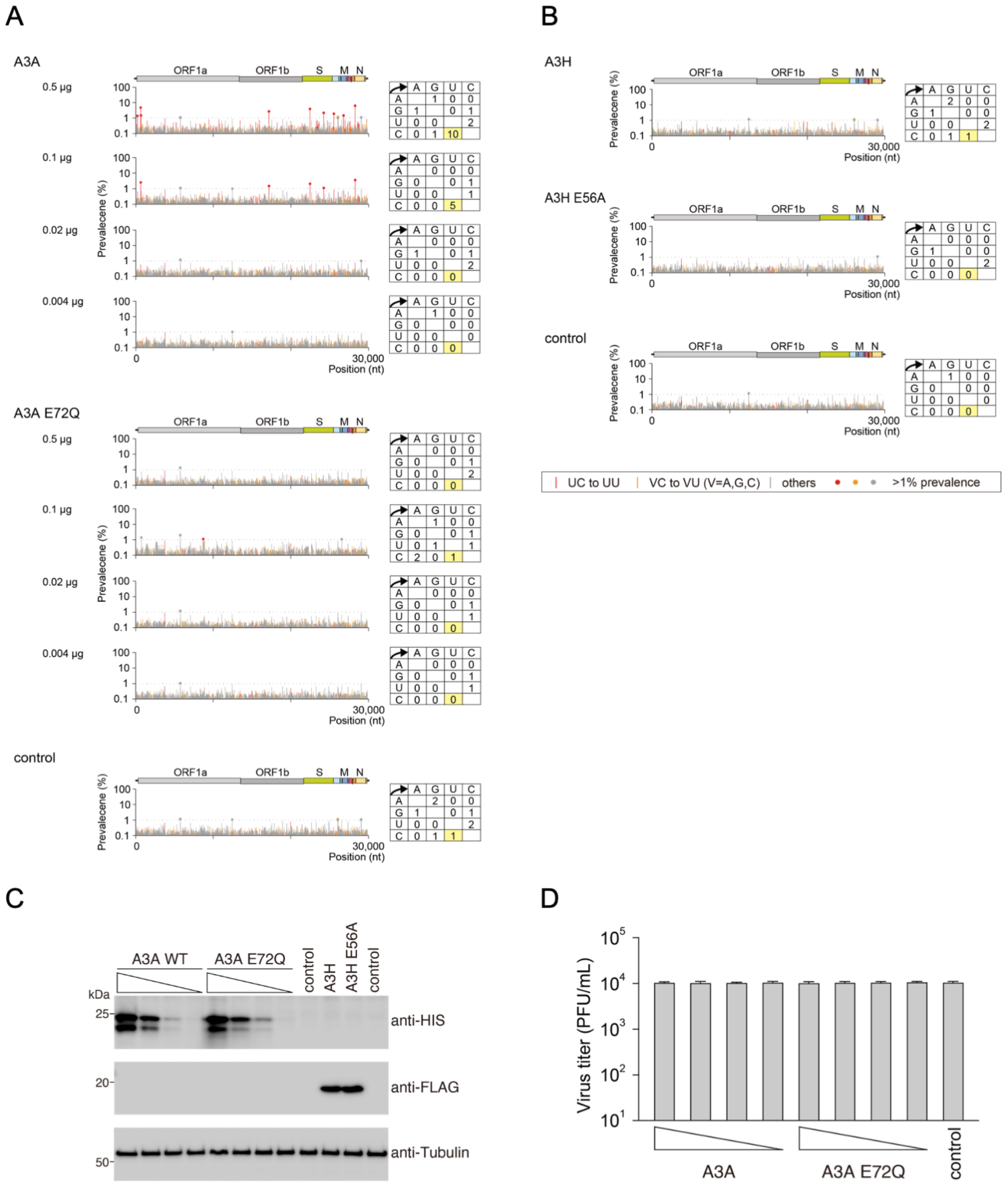

Supplementary Figure S5. The UC-to-UU mutation prevalence in the viral genome is increased depending on the expression levels of A3A, but not on those of A3A E72Q. (**A**) 293AT cells were transfected with different amounts of the expression plasmids (0.5, 0.1, 0.02 and 0.004 μg) for A3A or A3A E72Q. Empty vector was used as the negative control (control). Thirty-six hours after transfection, the cells were infected with SARS-CoV-2 (B.1.1) (MOI = 0.5). Twenty-four hours post-infection, cell culture supernatants were harvested. Viral genomes were sequenced on the Illumina MiSeq system. The prevalence of C-to-U mutations at different dinucleotide motifs was scored. The numbers of genomic positions where the prevalence of UC-to-UU, VC-to-VU (V = not U), and other C-to-U mutations was equal to or greater than 1% are shown. Mutation matrices indicate numbers of the substitution positions with a prevalence of ≥1% in each sample. (**B**) The effects of A3H and deaminase-defective A3H E56A on the prevalence of C-to-U mutations were also analysed in the same manner performed in the panel A, except for use of the A3H and A3H E56A plasmids (0.5 μg). The prevalence of the C-to-U mutations was comparable between A3H

and A3H E56A. This result suggest that A3H deaminase activity plays no critical roles in the induction of genomic UC-to-UU mutations in SARS-CoV-2 genome. (**C**) The expression levels of A3A, A3A E72Q, A3H and A3H E56A proteins in the transfected 293AT cells were analysed by immunoblotting with anti-HIS (for A3A) or anti-FLAG (for A3H) mAbs. Empty vector (Vector) was used as the negative control. An anti-ß-tubulin antibody was used as the loading control. (**D**) The viral titer (plaque-forming unit per mL: PFU/mL) in the cell culture supernatants was determined by plaque assay (Supplementary Methods). The data are presented as the means ± SDs for triplicate.

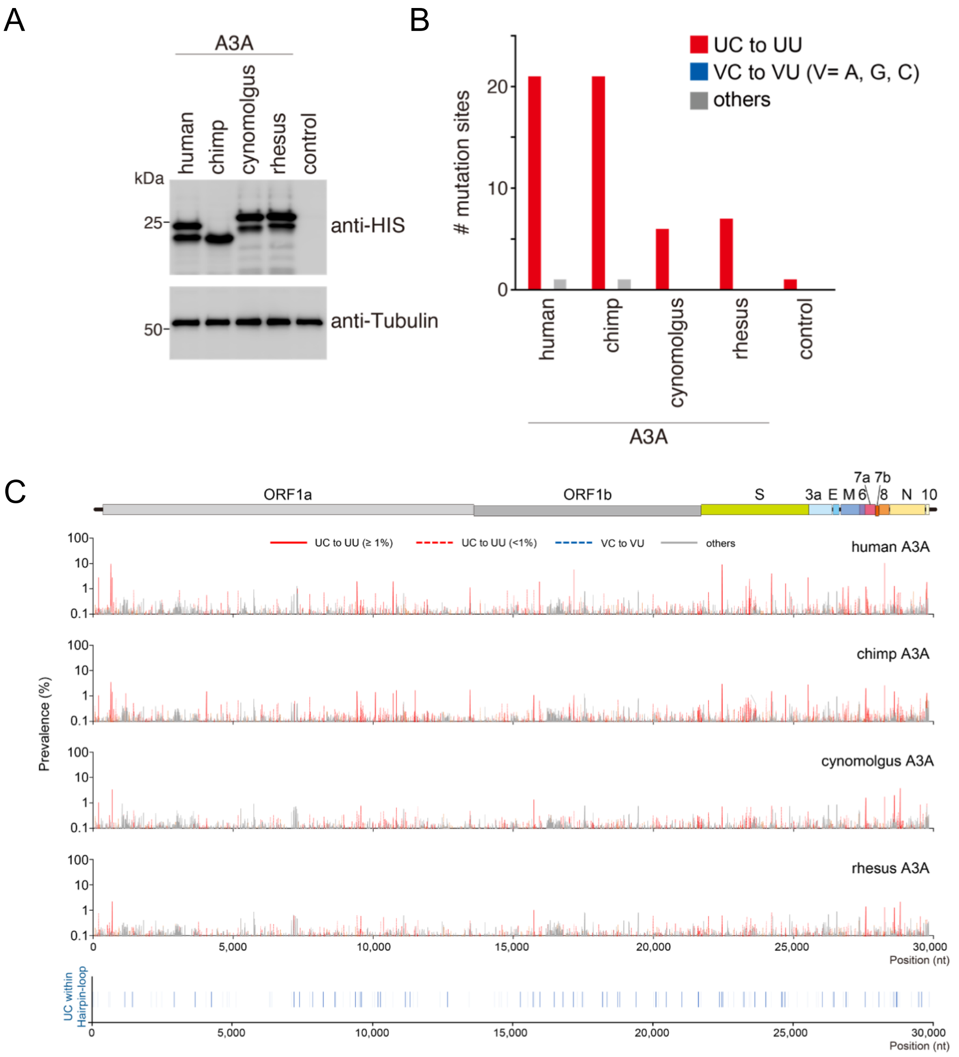

Supplementary Figure S6. Exogenous expression of primate A3A orthologues induces C-to-U editing in the SARS-CoV-2 genome. (**A**) Protein expression levels were compared between four primate A3As derived from *Homo sapiens* (human), *Pan troglodytes verus* (chimpanzee), *Macaca fascicularis* (cynomolgus macaque), and *Macaca mulatta* (rhesus macaque). 293AT cells were transfected with a C-terminal Myc-HIS-tagged A3A expression plasmid (Supplementary Methods). Intracellular A3A protein levels 36 h post-transfection were examined by immunoblotting using an anti-HIS tag mAb. A lower-molecular-weight band corresponding to human, cynomolgus, and rhesus A3A appeared in the products because of internal translation initiation at the second methionine (M13). Chimpanzee A3A was visualized as a single band, likely because its sequence does not contain a "first methionine (M1)" residue corresponding to human M1. An anti-ß-tubulin antibody was used as the loading control. (**B**) Transfected 293AT cells were infected with SARS-CoV-2 (B.1.1) (MOI = 0.5) at 36 h post-transfection. Twenty-four hours post-infection, the culture supernatants were collected for viral sequencing analysis on the Illumina MiSeq system. The numbers of genomic positions with UC-to-UU mutations (a prevalence of ≥ 1%) are shown in the bar graph. VC-to-VU (V = A, G or C) mutations (≥ 1%) were not detected. (**C**) The prevalence and genomic positions of the mutations induced by exogenous A3A expression were compared among the four primate A3A orthologues. The prevalence (%) of UC-to-UU mutations (red), VC-to-VU mutations (blue dotted line), and other mutations (grey line) at each position in the SARS-CoV-2 genome are shown. The solid and dotted red lines indicate a prevalence of ≥ 1% and < 1%, respectively.

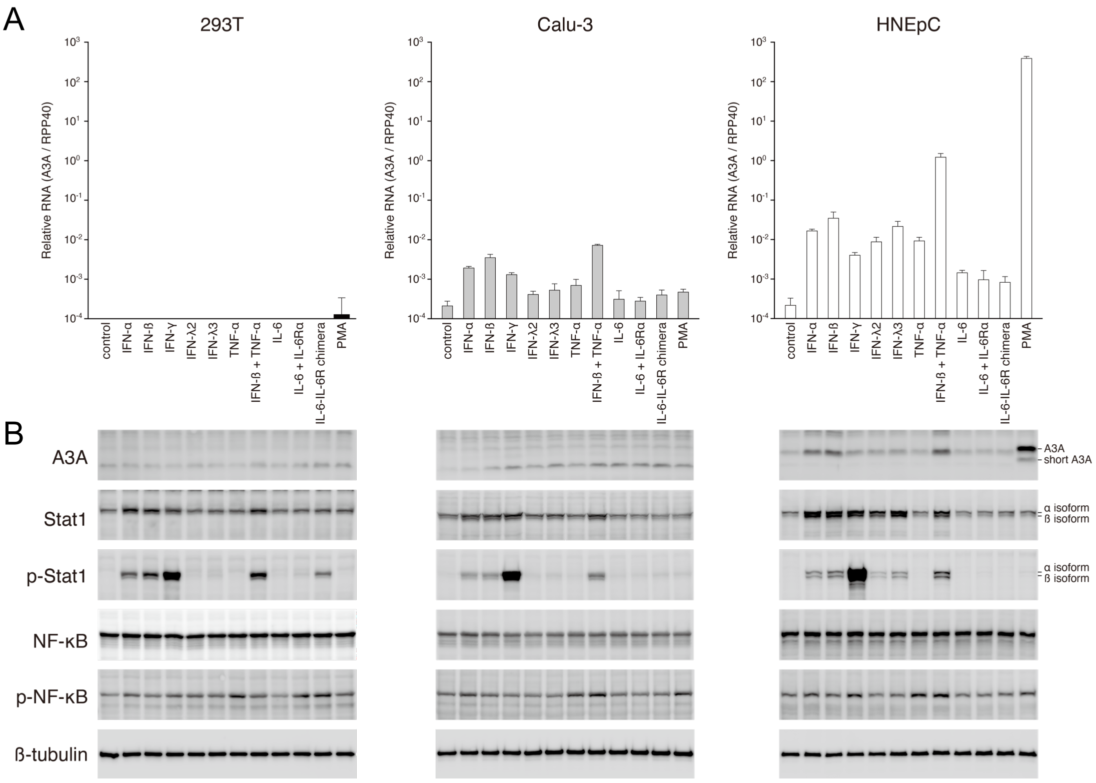

Supplementary Figure S7. Treatment with IFNs and proinflammatory cytokines induces A3A in Calu-3 cells and HNEpCs but not in 293T cells. (**A** and **B**) Cells were treated with IFN-α, IFN-ß, IFN-γ, INF-λ2, IFN-λ3, TNF-α, IFN-ß plus TNF-α, interleukin-6 (IL-6), IL-6 plus IL-6 receptor-α (IL-6Rα), an IL-6-IL6R chimaera, or PMA for 18 h (Supplementary Methods). (**A**) Using extracted cellular RNA, the mRNA copy numbers of A3A and the housekeeping gene RPP40 were quantified by quantitative RT–ddPCR. The A3A mRNA level relative to the RPP40 mRNA level was calculated. The data are presented as the means ± SDs (n = 3). (**B**) Protein levels of A3A, signal transducer and activator of transcription 1 (Stat1), tyrosine-phosphorylated Stat1 (phospho-Stat1, Tyr701), nuclear factor-kappa B (NF-κB) p65 and serine-phosphorylated NF-κB p65 (phospho-NF-κB, Ser536) were analysed by immunoblotting using anti-ApoC17 rabbit serum, anti-Stat1 rabbit serum, an anti-phospho-Stat1 (Tyr701) (58D6) rabbit mAb, an anti-NF-κB p65 (D14E12) XP rabbit mAb, and an anti-phospho-NF-κB p65 (Ser536) (93H1) rabbit mAb, respectively (all purchased from Cell Signaling Technology). An anti-ß-tubulin antibody was used as the loading control. Short A3A is an internal initiation product of translation initiation from the second methionine (M13).

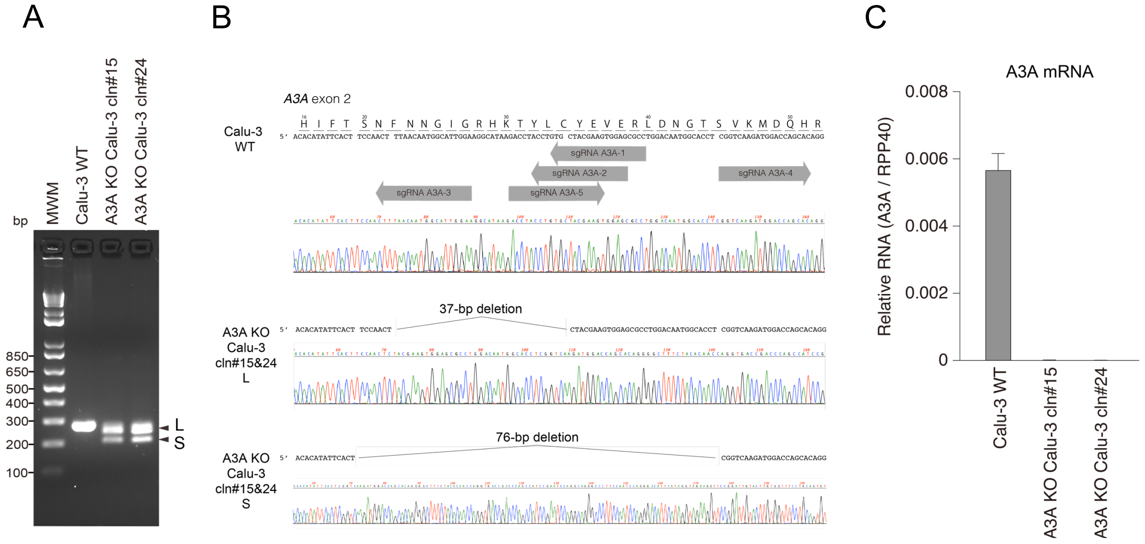

Supplementary Figure S8. Validation of A3A-KO Calu-3 cells generated by CRISPR/Cas9. (**A**) Two Calu-3 cell clones carrying deletions in the A3A nucleotide sequence (exon 2) were generated by transient transfection with a Cas9 expression plasmid and five single guide RNA (sgRNA) expression plasmids targeting the A3A gene. Genomic DNA fragments, including the five A3A-targeting guide sequences, were amplified by PCR, separated by 2% agarose gel electrophoresis and visualized by staining with ethidium bromide. The DNA fragments amplified from wild-type Calu-3 cells (Calu-3 WT) contained 284 base pairs (bps). Two A3A-KO cell clones, #15 and #24, produced two short fragments: longer (L) and shorter (S). (**B**) The PCR-amplified fragments were inserted into cloning vectors, and their DNA sequences were determined by Sanger sequencing. The panel shows the nucleotide and amino acid sequences of A3A exon 2 around the guide sequences (arrows). Representative electropherograms obtained by Sanger sequencing of the DNA sequences of alleles in Calu-3 WT cells and the L and S alleles in A3A-KO Calu-3 cells (clones #15 and #24) are shown. The L and S fragments had 37-bp and 76-bp deletions, respectively. These deletions generate frameshifts in the A3A coding sequence. (**C**) The mRNA copy number of A3A relative to that of RPP40 was analysed by RT-ddPCR. Total RNA isolated from each cell line after IFN-ß (1,000 U/mL) and/or TNF-α (50 ng/mL) treatment was used. No A3A mRNA expression was observed in A3A-KO cell clones #15 and #24. The data are presented as the means ± SDs (n = 3). For further analysis, clone #15 was used in this study.

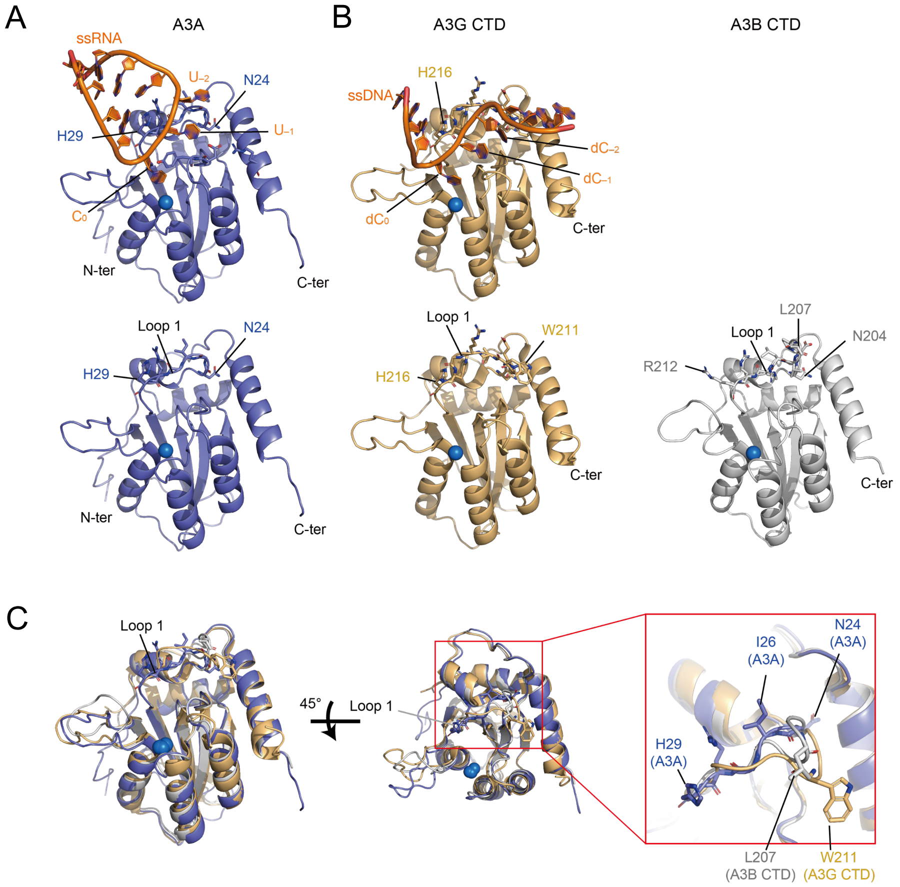

D

							Loop 1											
A3A	22	F	N	N	G	–	–	–	I	G	R	–	–	–	H	K	T	31
A3B	202	F	N	N	D	P	L	V	L	R	R	–	–	–	R	Q	T	214
A3C	21	F	K	N	L	W	E	A	N	D	R	–	–	–	N	E	T	33
A3D	217	F	K	N	L	L	K	A	C	G	R	–	–	–	N	E	S	229
A3F	204	F	K	N	L	R	K	A	Y	G	R	–	–	–	N	E	S	216
A3G	206	F	N	N	E	P	W	V	R	G	R	–	–	–	H	E	T	218
A3H	13	F	N	N	K	R	R	L	R	R	P	Y	Y	P	R	K	A	28

Supplementary Figure S9. The loop 1 structures of the A3B and A3G CTDs hinder the recognition of ssRNA for deamination. (**A**) An interaction of A3A (navy) with an RNA stem loop (orange) was predicted by in silico structural modelling. The deamination target cytidine (C_0) and flanking uridines (U_{-1} and U_{-2}) are key nucleotides in the substrate trinucleotide motif. (**B**) The crystal structure of the A3G CTD (wheat) bound to ssDNA (Protein Data Bank [PDB] ID 6BUX) (orange) and a predicted structure of the A3B CTD (grey) are shown. The loop 1 residues are highlighted with sticks. Zinc ions at the catalytic centres are indicated by blue spheres. The deamination target deoxycytidine (dC_0) and flanking deoxycytidines (dC_{-1} and dC_{-2}) are key nucleotides in the substrate trinucleotide motif. (**C**) A superposition of the structures of A3A, the A3B CTD, and the A3G CTD is shown, highlighting their conformations of loop 1. A3A N24-H29, A3B CTD L207, and A3G CTD W211 are indicated as sticks. Bulky loop 1 regions in the A3G and A3B CTDs protrude into the substrate binding pockets for pyrimidine (–1). In particular, A3B L207 and the A3G W211 form structural hindrances to the accommodation of ssRNA but not ssDNA. (**D**) The amino acid sequence of A3A loop 1 is 3 residues shorter than those of the A3B and A3G CTDs. Sequence alignment of the reference sequences of human A3A (GenBank ID NM_145699.4), A3B CTD (NM_004900.5), A3C (NM_014508.3), A3D CTD (NM_152426.4), A3F CTD (NM_145298.6), A3G CTD (NM_021822.4), and A3H (NM_181773.5) were performed. Amino acid residues between the 22nd and 31st positions in A3A and their corresponding residues in the other A3s are shown. Highly conserved residues are highlighted with a grey background.

Supplementary Table S1 The open reading frame sequences in primate A3A orthologues

Name	Open reading frame sequence (5' to 3')
Chimpanzee A3A-Myc-HIS	ATGGACCCACACATATTCACTTCCAACTTTAACAACGGCATTGGAAGGCGTAAGACCT<br>ACCTGTGCTACGAAGTGGAGCGCCTGGACAATGGCACCTCGGTCAAGATGGACCAGCA<br>CAGGGGCTTTCTACACAACCAGGCTAAGAATCTTCTCTGTGGCTTTTACGGCCGCCAT<br>GCGGAGCTGTGCTTCTTGGACCTGGTTCCTTCTTTGAAGTTGGACCCGGCCCAGATCT<br>ACAGGGTCACTTGGTTCATCTCCTGGAGCCCCTGCTTCTCCTGGGGCTGTGCCGGGCA<br>AGTGCGTGCGTTCCTTCAGGAGAACACACACGTGAGACTGCGCATCTTCGCTGCCCGC<br>ATCTATGATTACGACCCCCTATATAAGGAGGCGCTGCAAATGCTGCGGGATGCTGGGG<br>CCCAAGTCTCCATCATGACCTACGATGAATTTAAGCACTGCTGGGACACCTTTGTGGA<br>CCACCAGGGATGTCCCTTCCAGCCCTGGGATGGACTAGAGGAGCACAGCCAAGCCCTG<br>AGTGGGAGGCTGCGGGCCATTCTCCAGAATCAGGGAAACAAGCTTGGGCCCGAACAAA<br>AACTCATCTCAGAAGAGGATCTGAATAGCGCCGTCGACCATCATCATCATCATCATTG<br>A
Cynomolgus A3A-Myc-HIS	ATGGACGGCAGCCCAGCATCCAGGCCCAGACACTTGATGGATCCAGACACATTCACTT<br>TCAACTTTAACAATGACCTTTCGGTCCGTGGACGGCACCAGACCTACTTGTGCTACGA<br>GGTGGAGCGCCTGGACAATGGCACCTGGGTCCCGATGGACGAGCGCAGGGGCTTTCTA<br>TGCAACAAGGCTAAGAATGTTCCCTGTGGTGATTACGGCTGCCACGTGGAGCTGCGCT<br>TCCTGTGTGAGGTTCCTTCTTGGCAGTTGGACCCGGCCCAGACATACAGGGTCACTTG<br>GTTCATCTCCTGGAGCCCCTGCTTCAGGAGGGGCTGTGCCGGGCAAGTGCGTGCGTTC<br>CTTCAGGAGAACAAACACGTGAGACTGCGCATCTTCGCTGCCCGCATCTATGATTACG<br>ACCCCCTGTATCAGGAGGCACTGCGAACGCTGCGGGATGCTGGGGCCCAAGTCTCCAT<br>CATGACCTACGAGGAATTTAAGCACTGCTGGGACACCTTTGTGGACCGCCAGGGACGT<br>CCCTTCCAGCCCTGGGATGGACTAGATGAGCACAGCCAAGCCCTGAGTGGAAGGCTTC<br>GGGCCATTCTCCAGAATCAGGGAAACAAGCTTGGGCCCGAACAAAAACTCATCTCAGA<br>AGAGGATCTGAATAGCGCCGTCGACCATCATCATCATCATCATTGA
Rhesus A3A-Myc-HIS	ATGGACGGCAGCCCAGCATCCAGGCCCAGACACTTGATGGATCCAAACACGTTCACTT<br>TCAACTTTAACAATGACCTTTCGGTCCGTGGACGGCACCAGACCTACTTGTGCTACGA<br>GGTGGAGCGCCTGGACAATGGCACCTGGGTCCCGATGGACGAGCGCAGGGGCTTTCTA<br>CAAAACAAGGCTAAGAATGTTCCCTGTGGTGATTACGGCTGCCACGTGGAGCTGCGCT<br>TCCTGTGTGAGGTTCCTTCTTGGCAGTTGGACCCGGCCCAGACATACAGGGTCACTTG<br>GTTCATCTCCTGGAGCCCCTGCTTCAGGAAGGGCTGTGCCGGGCAAGTGCGTGCGTTC

	CTTCAGGAGAACAAACACGTGAGACTGCGCATCTTCGCTGCCCGCATCTATGATTACG<br>ACCCCCGGTATCAGGAGGCACTGCGAACGCTGCGGGATGCAGGGGCCCAAGTCTCCAT<br>CATGACCTACGAGGAATTTAAGCACTGCTGGGACACCTTTGTGGACCGCCAGGGACGT<br>CCCTTCCAGCCCTGGGATGGACTAGATGAGCACAGCCAAGCCCTGAGTGGAAGGCTTC<br>GGGCCATTCTCCAGAATCAGGGAAACAAGCTTGGGCCCGAACAAAAACTCATCTCAGA<br>AGAGGATCTGAATAGCGCCGTCGACCATCATCATCATCATCATTGA

Supplementary Table S2 Oligonucleotides used in this study

Primer Name	DNA sequence (5' to 3')
A3A(Pan.t.v)-100(+)	CCACGCCTTGAGCAAGTCGCA
A3A(Pan.v.t)1st(-)	CGTCTGTGAGCAGCTGGAGA
A3A(Pan.v.t)-4NotI(+)	TCGAGCGGCCGCCTTGATGGACCCACACATATTCACTTC
A3A (-) (NH) term-del	CGATACAAGCTTGTTTCCCTGATTCTGGAGAATGGC
A3A (Mac) 1st (+)	ATGGACGGCAGCCCAGCATCC
A3A (Mac) 1st (-)	CTTCCTTAGAGACTGAGGCCCATCCT
A3A (Mac) 2nd (+) NotI	TCGAGCGGCCGCCATGGACGGCAGCCCAGCATCCAGG
p-A3A E72Q (-)	5´-phospho-GCGCATGGCGGCCGTAAAAGCCACAGAG
p-A3A E72Q (+)	5´-phospho-AGCTGCGCTTCTTGGACCTGGTTCCTTCT
sgRNA-A3A_1F	5´-phospho-<br>GGGGCGCTCCACTTCGTAGCACGTTTTAGAGCTAGAAATAGCAAGTTAAAATAAGGC
sgRNA-A3A_2F	5´-phospho-GGCTCCACTTCGTAGCACAGGTGTTTTAGAGCTAGAAATAGCAAGTTAAAATAAGGC
sgRNA-A3A_3F	5´-phospho-GGTCCAATGCCATTGTTAAAGTGTTTTAGAGCTAGAAATAGCAAGTTAAAATAAGGC
sgRNA-A3A_4F	5´-phospho-GGCGGTCAAGATGGACCAGCACGTTTTAGAGCTAGAAATAGCAAGTTAAAATAAGGC
sgRNA-A3A_5F	5´-phospho-GGGACCTACCTGTGCTACGAAGGTTTTAGAGCTAGAAATAGCAAGTTAAAATAAGGC
sgRNA-A3A_1R	5´-phospho-GGTGTTTCGTCCTTTCCACAAGATATATAAAGCCAAG
A3A_Seq_for_Ex2_1(+)	GAGGCAGGGCAGTCCAGGA
A3A_KO_Seq_3(-)	GTACAATCCGGAACCTTCTACCTATG

SUPPLEMENTARY METHODS

Viruses and plasmids

The virus strains used in the experiments described in the extended data were SARS-CoV-2 QK002 (Alpha, Pango lineage B.1.1.7, GISAID EPI ISL 768526), SARS-CoV-2 TY8-612 (Beta, Pango lineage B.1.351, GISAID EPI ISL 1123289) and SARS-CoV-2 TY7-501 (Gamma, Pango lineage P.1, GISAID EPI ISL 833366) obtained from the National Institute of Infectious Diseases, Japan. The viruses were propagated in Vero E6 cells. The virus titres were determined using Vero E6/TMPRSS2 cells.

For construction of primate A3A expression plasmids, A3A cDNAs reverse transcribed from total mRNA samples of the chimpanzee (*Pan troglodytes*) (GenBank ID JN177348), the HSP-239 lymphocyte cell line, and peripheral blood lymphocytes (PBLs) of cynomolgus macaque (*Macaca fascicularis*) (ID MF54) (GenBank ID LC710897),and rhesus macaque (*Macaca mulatta*) (GenBank ID JF831054) were amplified by nested RT–PCR. HSP-239 cells were obtained from the Japanese Collection of Research Bioresources Cell Bank. Total mRNA from cynomolgus and rhesus macaque PBLs was generously provided by Dr Hirofumi Akari (Primate Research Institute, Kyoto University, Japan). RT–PCR was performed using a PrimeScript II High Fidelity One Step RT–PCR kit (TAKARA Bio) and the appropriate primer sets (listed in Supplementary Table S2): for chimpanzee A3A – 1st PCR, A3A(Pan.t.v)-100(+) and A3A(Pan.v.t)1st(-), and 2nd PCR, A3A(Pan.v.t)-4NotI(+), and A3A (-) (NH) term-del; for cynomolgus and rhesus A3As, 1st PCR, A3A (Mac) 1st (+), and A3A (Mac) 1st (-), and 2nd PCR, A3A (Mac) 2nd (+) NotI, and A3A (-) (NH) term-del. The amplified DNA fragments were replaced with the human A3A gene inserted between the NotI and HindIII sites in the pcDNA A3A wild-type (WT) (Myc-HIS) plasmid. The open reading frame sequences in chimpanzee, cynomolgus, and rhesus A3A are listed in Supplementary Table S1. To generate the A3A E72Q mutant expression plasmid, a single substitution was introduced by oligonucleotide-directed PCR using the pcDNA A3A WT (Myc-HIS) plasmid and a primer set (p-A3A E72Q (+) and p-A3A E72Q (-)) (Supplementary Table S2). The sequences of both the inserts and boundary regions of all plasmids were verified by Sanger sequencing. Notably, human, cynomolgus, and rhesus A3As often have an additional product derived from internal translation initiation at the second methionine (M13). Because chimpanzee A3A has no AUG start codon corresponding to the codon beginning with the first methionine (M1) in humans (and therefore no N-terminal 12-amino acid truncation), a single A3A protein product was translated from M13 in chimpanzee-derived cells (1).

IFN and proinflammatory cytokine treatments

293T cells, Calu-3 cells, and HNEpCs cultured in 12-well plates were treated with IFN-α (1,000 U/mL) (Universal Type I IFN, PBL Assay Science), IFN-ß (1,000 U/mL), IFN-γ (100 ng/mL) (Pepro Tech Inc.), IFN-λ2 (20 ng/mL) (R&D Systems), IFN-λ3 (2,000 U/mL) (PBL Assay Science), TNF-α (50 ng/mL), IFN-ß plus TNF-α, IL-6 (50 ng/mL) (Pepro Tech Inc.), IL-6 (50 ng/mL) plus soluble IL-6 receptor-α (IL-6Rα) (100 ng/mL) (R&D systems), an IL-6-IL6R chimaera (100 ng/mL) (R&D systems), or PMA (100 nM) (Sigma–Aldrich) in 5% CO_2 for 18 h at 37°C. The cells were harvested to extract total mRNA and to prepare total cellular protein for immunoblotting. To quantify the mRNA copy numbers of A3A and RPP40, quantitative reverse transcription–droplet digital PCR (RT–ddPCR) was performed. The ratio of the A3A mRNA copy number to the RPP40 mRNA copy number was calculated. To analyse protein expression, immunoblotting was performed using the appropriate primary antibodies. Anti-ApoC17 rabbit serum ($1:1\times10^3$ dilution) (AIDS Research and Reference Reagent Program) was used to detect endogenous A3A. This monoclonal antibody (mAb) cross-reacts with A3A and A3G, which are distinguishable in SDS–PAGE gels, as previously reported (2). Anti-Stat1 rabbit serum, an anti-phosphorylated (phospho)-Stat1 (Tyr701) (58D6) rabbit mAb, an anti-NF-κB p65 (D14E12) XP rabbit mAb, and an anti-phospho-NF-κB p65 (Ser536) (93H1) rabbit mAb (all purchased from Cell Signaling Technology) were used to detect Stat1, phospho-Stat1 (Tyr701), NF-κB, and phospho-NF-κB (Ser536), respectively (all at a $1:1\times10^3$ dilution). Horseradish peroxidase (HRP)-conjugated anti-rabbit IgG ($1:2\times10^4$ dilution) was used as the secondary antibody. Proteins were visualized by enhanced chemiluminescence using SuperSignal West Dura substrate (Thermo Fisher Scientific) and an ImageQuant LAS 4000 system (GE Healthcare Life Sciences).

Generation of A3A-KO Calu-3 cells

Cells with CRISPR/Cas9-based A3A KO were generated as previously reported by Cortez et al. (3), with slight modifications. In brief, 5´-phosphate oligonucleotides (Supplementary Table S2) containing the guide sequences were synthesized (Fasmac Co.). The single guide RNA (sgRNA) expression plasmid, pGL3-U6-sgRNA-PGK-puromycin (Addgene), which was digested with AgeI, was amplified by PCR using five sets of 5´-phosphate oligonucleotides: forward primers, sgRNA-A3A_1F, sgRNA-A3A_2F, sgRNA-A3A_3F, sgRNA-A3A_4F, and sgRNA-A3A_5F; and a common reverse primer, sgRNA-A3A_1R. Amplified plasmid DNAs (~4,975 base pairs) were self-ligated with T7 DNA Ligase (New England Biolabs) and were then cloned using *E. coli*. The sequences of both the sgRNA sequence and boundary regions of all plasmids were verified by Sanger sequencing. Next, the five sgRNA expression plasmids and pST1374-NLS-flag-linker-cas9 (Addgene) were cotransfected into Calu-3 cells using FuGENE HD. Seventy-two hours

posttransfection, the cells were transiently selected by the addition of 2 μg/mL puromycin (Qbiogene) and 15 μg/mL blasticidin S hydrochloride (Fujifilm Wako Pure Chemical Co.) to the cell culture medium. After measuring the cell concentration, surviving cells were isolated by limiting dilution and propagated for approximately two months. To confirm A3A KO, cloned A3A-KO Calu-3 cells were subjected to genomic DNA purification using the DNeasy Blood & Tissue Kit (QIAGEN). First, DNA fragments containing the five A3A-targeting guide sequences were amplified by PCR using a set of primers (A3A_Seq_for_Ex2_1(+) and A3A_KO_Seq_3(-)) (Supplementary Table S2) prior to separation by electrophoresis on a 2% agarose gel and visualization by staining with ethidium bromide (Sigma–Aldrich). Cloned cells with reduced sizes of the DNA fragments were selected for further confirmation of A3A KO. The fragments were cloned into the pUC118 plasmid vector using the Mighty Cloning Reagent Set (Takara Bio), and their DNA sequences were determined by Sanger sequencing using the primer A3A_Seq_for_Ex2_1(+) (Supplementary Table S2). Notably, it was technically difficult to determine the A3A genome sequences by the Sanger method using sequencing primers that bind to the upstream region (the 3´ region of the 1st intron in A3A) of the primer A3A_Seq_for_Ex2_1(+), partially because this region of the A3A genome is highly G-rich. Clones #15 and #24 of A3A-KO Calu-3 cells exhibited a similar type of large A3A genomic deletion (in the 2nd exon) by CRISPR/Cas9 gene editing. We used clone #15 in this study for further analysis.

in silico structural analysis

The structure of the A3A-RNA complex was initially modelled based on a crystal structure of A3A-ssDNA (Protein Data Bank [PDB] ID 5SWW) (4) with Modeller 9v8 (5). In our model, the RNA sequence identified at position "X" in Figure 2C was used. To stabilize the hairpin loop structure of the RNA, the distances between the base pairs in RNA stem were set to be constrained with a Gaussian function. Next, unfavourable contacts and interactions within the initial structure were relaxed by molecular dynamics (MD) simulation on a 50.0 ns timescale using the Amber16 software package (http://ambermd.org/) (6), as reported previously by our group (7). In the MD simulation, the base pairs of the RNA hairpin loop were constrained with a harmonic potential of 1.0 kcal/mol/$Å^2$ against base pairs with distances longer than 4.0 Å. The final trajectory was selected as the predicted structure in this study. For modelling of the A3B CTD structure, homology modelling was performed based on a crystal structure of the A3B CTD variant (PDB ID 5TD5) (4) using Modeller 9v8, because the variant determined previously has a loop 1 sequence different from that of the WT A3B CTD (GenBank ID NM_004900.5).

Viral titer determination

Plaque assay was performed using VeroE6/TMPRSS2 cells to determine viral titer according to the detailed protocol as previously reported (8). Briefly, the cell culture supernatant containing live SARS-CoV-2 was ten-fold serially diluted and inoculated onto the cells in 12-well culture plates. The cells were incubated for 1 h at 37°C, rocking every 15 min. After wash with DMEM GM, 1.5% carboxymethylcellulose (Sigma-Aldrich) in Minimum Essential Media (Thermo Fisher Scientific) containing 3.5% FBS, 1.8 mM L-glutamine (Thermo Fisher Scientific) and nonessential amino acid solution (Thermo Fisher Scientific, cat#11140050) was overlaid and then incubated for 3 d at 37°C in a 5% CO_2 incubator. After careful removal of the overlaid solution, the cells were gently washed with DMEM GM once, fixed with 10% formalin (Fujifilm Wako Pure Chemical Co.) for 1 h, and stained with 1% Crystal violet solution (Bio Medical Science). The numbers of plaques observed per well at each dilution were counted.

References for Supplementary Methods

1. Henry, M., Terzian, C., Peeters, M., Wain-Hobson, S. and Vartanian, J.P. (2012) Evolution of the primate APOBEC3A cytidine deaminase gene and identification of related coding regions. *PLoS One*, **7**, e30036.
2. Goila-Gaur, R., Khan, M.A., Miyagi, E., Kao, S. and Strebel, K. (2007) Targeting APOBEC3A to the viral nucleoprotein complex confers antiviral activity. *Retrovirology*, **4**, 61.
3. Cortez, L.M., Brown, A.L., Dennis, M.A., Collins, C.D., Brown, A.J., Mitchell, D., Mertz, T.M. and Roberts, S.A. (2019) APOBEC3A is a prominent cytidine deaminase in breast cancer. *PLoS Genet*, **15**, e1008545.
4. Shi, K., Carpenter, M.A., Banerjee, S., Shaban, N.M., Kurahashi, K., Salamango, D.J., McCann, J.L., Starrett, G.J., Duffy, J.V., Demir, O. *et al.* (2017) Structural basis for targeted DNA cytosine deamination and mutagenesis by APOBEC3A and APOBEC3B. *Nat Struct Mol Biol*, **24**, 131-139.
5. Sali, A. and Blundell, T.L. (1993) Comparative protein modelling by satisfaction of spatial restraints. *J Mol Biol*, **234**, 779-815.
6. Case, D.A., Cheatham, T.E., 3rd, Darden, T., Gohlke, H., Luo, R., Merz, K.M., Jr., Onufriev, A., Simmerling, C., Wang, B. and Woods, R.J. (2005) The Amber biomolecular simulation programs. *J Comput Chem*, **26**, 1668-1688.
7. Matsuoka, T., Nagae, T., Ode, H., Awazu, H., Kurosawa, T., Hamano, A., Matsuoka, K., Hachiya, A., Imahashi, M., Yokomaku, Y. *et al.* (2018) Structural basis of chimpanzee

APOBEC3H dimerization stabilized by double-stranded RNA. *Nucleic Acids Res*, **46**, 10368-10379.

8. Mendoza, E.J., Manguiat, K., Wood, H. and Drebot, M. (2020) Two Detailed Plaque Assay Protocols for the Quantification of Infectious SARS-CoV-2. *Curr Protoc Microbiol*, **57**, ecpmc105.
